# The Impact of p53 on Aristolochic Acid I-Induced Gene Expression In Vivo

**DOI:** 10.3390/ijms20246155

**Published:** 2019-12-06

**Authors:** Mateja Sborchia, Hector C. Keun, David H. Phillips, Volker M. Arlt

**Affiliations:** 1 Department of Analytical, Environmental and Forensic Sciences, MRC-PHE Centre for Environment and Health, King’s College London, London SE1 9NH, UK; mateja.sborchia@kcl.ac.uk (M.S.); david.phillips@kcl.ac.uk (D.H.P.); 2 Department of Surgery and Cancer, Faculty of Medicine, Imperial College London, London W12 0NN, UK; h.keun@imperial.ac.uk

**Keywords:** aristolochic acid I, tumour suppressor p53, mouse models, carcinogen metabolism, microarray, gene expression

## Abstract

Exposure to aristolochic acid (AA) is linked to kidney disease and urothelial cancer in humans. The major carcinogenic component of the AA plant extract is aristolochic acid I (AAI). The tumour suppressor p53 is frequently mutated in AA-induced tumours. We previously showed that p53 protects from AAI-induced renal proximal tubular injury, but the underlying mechanism(s) involved remain to be further explored. In the present study, we investigated the impact of p53 on AAI-induced gene expression by treating *Trp53(+/+)*, *Trp53(+/-),* and *Trp53(-/-)* mice with 3.5 mg/kg body weight (bw) AAI daily for six days. The Clariom™ S Assay microarray was used to elucidate gene expression profiles in mouse kidneys after AAI treatment. Analyses in Qlucore Omics Explorer showed that gene expression in AAI-exposed kidneys is treatment-dependent. However, gene expression profiles did not segregate in a clear-cut manner according to *Trp53* genotype, hence further investigations were performed by pathway analysis with MetaCore™. Several pathways were significantly altered to varying degrees for AAI-exposed kidneys. Apoptotic pathways were modulated in *Trp53(+/+)* kidneys; whereas oncogenic and pro-survival pathways were significantly altered for *Trp53(+/-)* and *Trp53(-/-)* kidneys, respectively. Alterations of biological processes by AAI in mouse kidneys could explain the mechanisms by which p53 protects from or p53 loss drives AAI-induced renal injury in vivo.

## 1. Introduction

The p53 transcription factor regulates numerous cellular processes, including DNA repair, apoptosis, cell cycle arrest, and metabolism [1]. More than 50% of human cancers are characterised by deregulations in *TP53* [2,3]. The critical role played by p53 in tumour suppression is delineated by *Trp53*(-/-) mice that develop cancers with complete penetrance [4,5]. Moreover, exposures to chemicals in the environment have been linked to characteristic *TP53* mutational patterns in human tumours [6].

**Figure 1:**
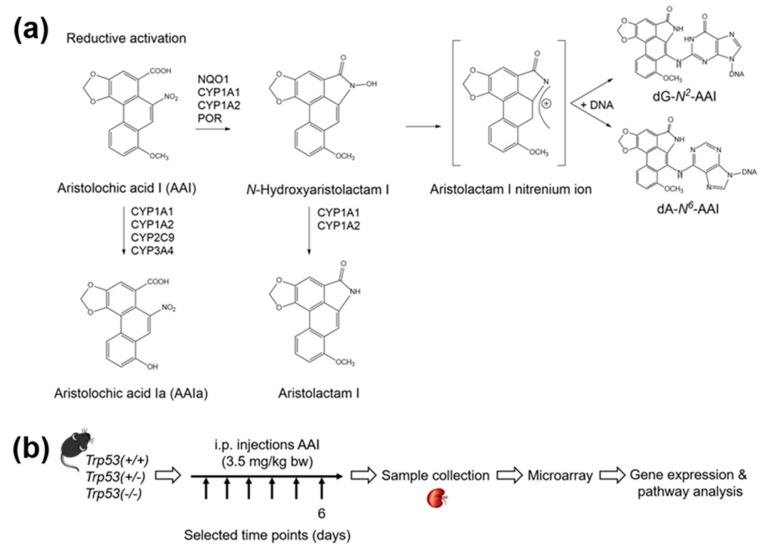
(**a**) Metabolic activation and detoxication pathways of aristolochic acid I (AAI). CYP: Cytochrome P450; dA-*N^6^*-AAI: 7-(deoxyadenosin-*N^6^*-yl)aristolactam I; dG-*N^2^*-AAI: 7-(deoxyguanosin-*N^2^*-yl)aristolactam I; NQO: NAD(P)H:quinone oxidoreductase; POR: NADPH:cytochrome P450 oxidoreductase. (**b**) Schematic representation of experimental design. *Trp53(+/+)*, *Trp53(+/-),* and *Trp53(-/-)* mice (*n* = 5/group) were treated with 3.5 mg/kg body weight (bw) AAI by intraperitoneal injection (i.p.) daily for 6 days. Controls were injected with water only. Kidneys were collected after six days of AAI treatment. The Clariom™ S Assay was used as a microarray platform. Gene expression and pathway analysis were conducted with Qlucore Omics Explorer and MetaCore™ software, respectively.

The environmental carcinogen aristolochic acid (AA) is present in *Aristolochia* plants which are used in medicinal herbal remedies worldwide [7,8]. The nitrophenanthrene carboxylic acid structure of AAI, which is the main component of the plant extract AA, is shown in Figure 1a [9,10]. Exposure to AA leads to particular DNA adducts that form as a result of AAI bioactivation by several enzymes, such as NAD(P)H:quinone oxidoreductase (NQO1) and cytochrome P450 (CYP) 1A1 and 1A2 (i.e., CYP1A1 and CYP1A2) (Figure 1a) [11,12,13,14]. The renal diseases aristolochic acid nephropathy (AAN) and Balkan endemic nephropathy (BEN) are both caused by AA exposure [8,15,16,17]. Furthermore, renal injury in AA-exposed individuals can lead to the development of upper urinary tract and bladder urothelial tumours, as well as renal cell carcinomas [18,19,20,21,22]. *Aristolochia*-containing herbal products have been banned in many countries around the world but their use continues and remains an issue for public health, particularly in Asia [8,23].

Exposure to AA is associated with characteristic AT to TA transversions, mutations frequently observed in *TP53* in both human tumours and experimental cell culture models [24,25,26,27]. AA also affects gene expression profiles and *TP53*-dependent pathways in vitro and in vivo [28,29,30]. Given the clear link between AA exposure and p53, it is of importance to study the role of this gene in AAI tumourigenesis. Previous work on kidneys isolated from AAI-treated (5 mg/kg bw daily for three, 12, or 21 days) *TP53(+/+)* Hupki (human *TP53* knock-in) mice demonstrated that AAI modulates the expression of genes that play a role in the cell cycle, stress response, immune system, inflammatory response, apoptosis, and kidney development [29]. Another study in rats treated with AA (10 mg/kg bw) also observed alterations in genes related to the defence response, immune response, and apoptosis [30]. Both studies [29,30] demonstrated that AA-induced changes in gene expression are tissue-specific, meaning alterations at the gene level occurred only in the kidney and not in the liver of AA-treated rodents.

Recent work on *Trp53(+/+)*, *Trp53(+/-),* and *Trp53(-/-)* mice in our group demonstrated that wild-type *Trp53* protects from AAI-induced nephrotoxicity [31]. Proximal tubular damage induced by 3.5 mg/kg bw AAI (daily treatment of six days) was higher in *Trp53(-/-)* kidneys than in *Trp53(+/+)* kidneys [31]. A role for p53 in AAI bioactivation was not observed as *Trp53* status did not impact on AAI-induced DNA adduct formation in vivo [31]. Thus, the underlying mechanism(s) by which *Trp53* impacts on AAI-induced nephrotoxicity remains to be further explored. Transcriptomic analysis can provide information on such mechanism(s), helping to define relationships between toxicological end-points and gene expression patterns, and predict toxic responses. In the present study, we explored gene expression changes by microarray technology in *Trp53(+/+)*, *Trp53(+/-),* and *Trp53(-/-)* kidneys derived from mice that were treated with AAI on the basis of a previously established protocol to study experimental AAN (Figure 1b).

## 2. Results

### 2.1. Gene Expression Analysis

Gene expression analysis was based on two major questions:

1. Which genes and pathways are modulated by AAI treatment in kidneys of *Trp53(+/+)*, *Trp53(+/-)*, and *Trp53(-/-)* mice?
2. Which genes and pathways are commonly and differentially altered between AAI-exposed *Trp53(+/+)*, *Trp53(+/-)*, and *Trp53(-/-)* kidneys?

**Figure 2:**
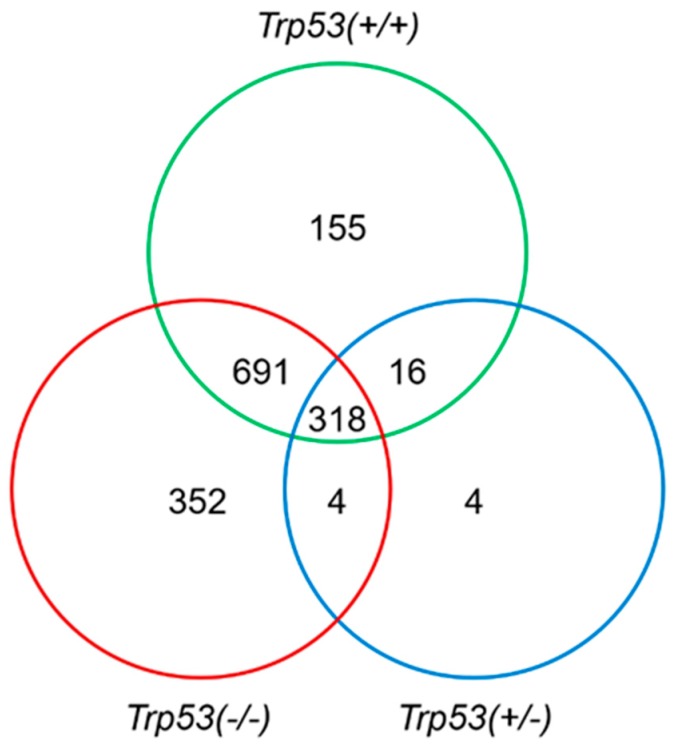
Gene content comparison for AAI-exposed *Trp53(+/+)*, *Trp53(+/-),* and *Trp53(-/-)* kidneys. Venn diagrams show genes whose expression was significantly altered (*p* < 0.05; fold change ± 2).

After applying the analysis parameters (*p* < 0.05; fold change ± 2), the fold change in gene expression relative to controls was obtained for AAI-exposed *Trp53(+/+)*, *Trp53(+/-),* and *Trp53(-/-)* kidneys (i.e., three separate gene lists were generated) using Qlucore Omics Explorer. A total of 1180 (↑ 653, ↓ 527), 342 (↑ 159, ↓ 183), and 1365 (↑ 737, ↓ 628) genes were up (↑)- or down (↓)-regulated in kidneys of *Trp53(+/+)*, *Trp53(+/-),* and *Trp53(-/-)* mice after AAI treatment, respectively. These are depicted in the Venn diagram shown in Figure 2.

To investigate similarities and differences in gene expression, the three aforementioned gene lists were directly compared using Qlucore Omics Explorer. A total of 846 (↑ 500, ↓ 346), 171 (↑ 126, ↓ 45), and 20 (↑ 14, ↓ 6) genes differed between kidneys of AAI-treated *Trp53(+/+)* and *Trp53(+/-)* mice; between *Trp53(+/+)* and *Trp53(-/-)* mice; and between *Trp53(+/-)* and *Trp53(-/-)* mice, respectively (Supplementary Table S1). These three gene lists are referred to as “differences”. In addition, a total of 334 (↑ 148, ↓ 186), 1009 (↑ 522, ↓ 487), and 322 (↑ 139, ↓ 183) genes were similar between kidneys of AAI-treated *Trp53(+/+)* and *Trp53(+/-)* mice; between *Trp53(+/+)* and *Trp53(-/-)* mice; and between *Trp53(+/-)* and *Trp53(-/-)* mice, respectively. These three gene lists are referred to as “intersections” (Supplementary Table S1).

The impact of *Trp53* genotype on AAI-induced gene expression in vivo was further investigated by comparing gene lists for AAI-exposed *Trp53(+/+)* (1180 genes), *Trp53(+/-)* (342 genes), and *Trp53(-/-)* (1365 genes) kidneys in MetaCore™. Significantly altered pathways (*p* < 0.05) were mapped by using the “Compare Experiments Workflow” tool, which provides information on intersections between gene lists. These include the number of genes that are in common and unique between gene lists. A total of 318 genes were in common for *Trp53(+/+)*, *Trp53(+/-),* and *Trp53(-/-)* kidneys (Figure 2), whereas a number of genes were genotype-specific. More precisely, 155, 4, and 352 genes were found to be uniquely altered in *Trp53(+/+)*, *Trp53(+/-),* and *Trp53(-/-)* kidneys, respectively (Figure 2).

### 2.2. The Impact of Trp53 Genotype on AAI-Induced Gene Expression In Vivo

Principle component analysis (PCA) was used to create a two-dimensional representation of the data set by illustrating differences in global gene expression profiles, whereas hierarchical clustering was applied to build a dendrogram that clusters samples according to similarities in gene expression. Both methods are unsupervised, meaning that prior knowledge on sample information is not utilised to create graphical representations.

**Figure 3:**
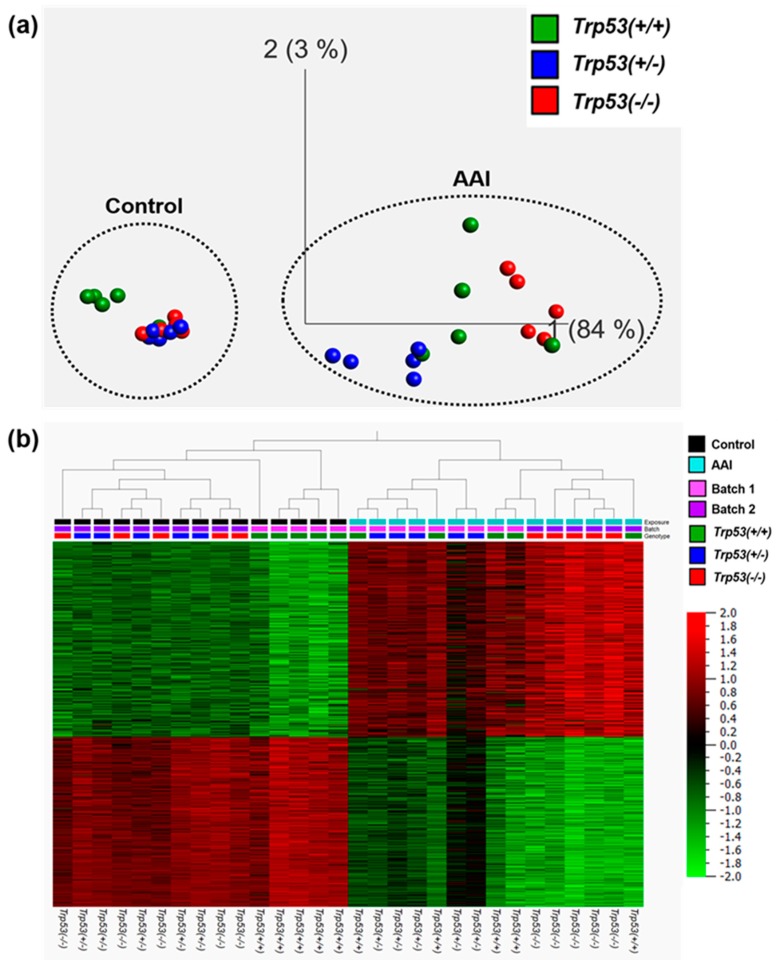
The impact of *Trp53* genotype on AAI-induced gene expression in vivo. (**a**) Principle component analysis (PCA) and (**b**) hierarchical clustering of significantly altered (*p* < 0.05; fold change ± 2) genes in kidneys of *Trp53(+/+)*, *Trp53(+/-),* and *Trp53(-/-)* mice. The heat map colours are based on gene expression (ordered in a decreasing manner for the AAI group), with red being upregulated and green being downregulated. Batches #1 and #2 indicate grouping of samples during cDNA synthesis.

Prior to investigating the impact of *Trp53* status on AAI-induced gene expression, significantly altered genes (*p* < 0.05; fold change ± 2) for AAI-exposed *Trp53(+/+)*, *Trp53(+/-),* and *Trp53(-/-)* kidneys were visually compared in Qlucore Omics Explorer. As shown in the PCA plot (Figure 3a), AAI treatment was the driving factor (84%) for gene expression. Overall, gene expression profiles clearly showed a treatment-dependent separation. This finding was further supported through hierarchical clustering (Figure 3b), which showed that control and AAI-exposed samples clustered separately.

Given that AAI treatment clearly had an effect on gene expression, the impact of *Trp53* genotype on AAI-induced gene expression was further examined. PCA analysis indicated some sort of *Trp53* genotype-dependent separation of the global gene expression profiles (Figure 3a). Colour intensities in the heat map (Figure 3b) indicated that control *Trp53(+/+)* kidneys separate from control *Trp53(+/-)* and *Trp53(-/-)* kidneys, whereas AAI-exposed *Trp53(-/-)* kidneys separate from AAI-exposed *Trp53(+/+)* and *Trp53(+/-)* kidneys, thus indicating a *Trp53* genotype-dependent effect on gene expression. However, these observations were not clear-cut and required further investigations through pathway analysis.

The effects of *Trp53* genotype and AAI treatment on gene expression were also explored visually on an individual basis in Qlucore Omics Explorer. Separate hierarchical clustering on significantly altered genes (*p* < 0.05; fold change ± 2) for *Trp53(+/+)*, *Trp53(+/-),* and *Trp53(-/-)* kidneys was carried out. As shown in Figure 3b, separate heat maps for *Trp53(+/+)*, *Trp53(+/-),* and *Trp53(-/-)* kidneys again showed a treatment-dependent separation (Supplementary Figure S1). All of the conducted analyses and obtained gene lists were corrected for batch effects with a built-in algorithm (Qlucore Omics Explorer).

### 2.3. Genes Modulated by AAI Treatment in Trp53(+/+), Trp53(+/-), and Trp53(-/-) Kidneys

**Figure 4:**
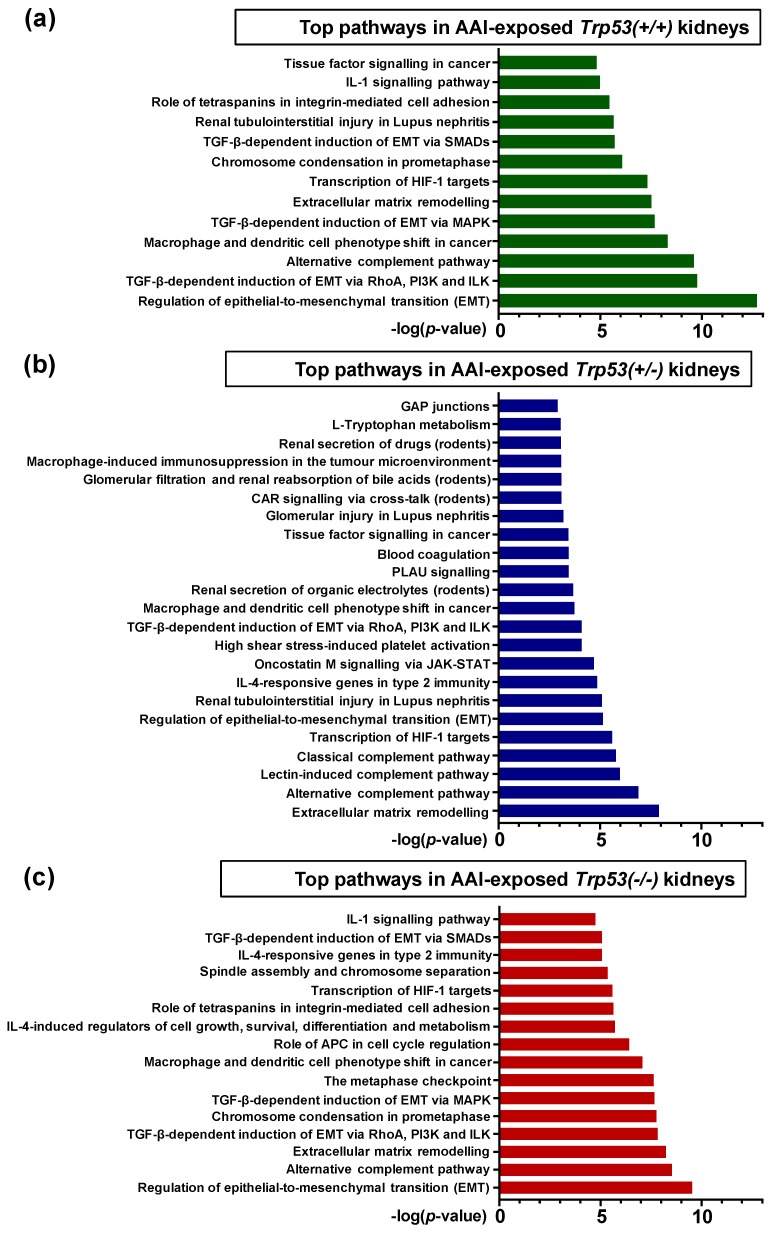
Top (**a**) 13, (**b**) 23, and (**c**) 16 pathways in AAI-exposed *Trp53(+/+)*, *Trp53(+/-),* and *Trp53(-/-)* kidneys, respectively. The significance of the pathways are shown by the -log(*p*-value). Relevant pathways were selected out of top 50 pathways (*p* < 0.05). A brief pathway description, statistical significance (*p*-value and FDR), and the number of genes found within the pathway, including a list of these, are shown in Table S3. Analysis was carried out with MetaCore™.

To investigate the role of *Trp53* genotype on AAI-induced gene expression in vivo, the online pathway analysis tool MetaCore™ was used. Gene lists for AAI-exposed *Trp53(+/+)* (1180 genes), *Trp53(+/-)* (342 genes), and *Trp53(-/-)* (1365 genes) kidneys were individually analysed. The enrichment method used in MetaCore™ (i.e., “Enrichment analysis in Pathway Maps”) maps the genes in the experiment to MetaCore™ ontologies, ultimately mapping the statistically significant pathways (*p* < 0.05) and obtaining the top fifty pathways for each gene list. A summary of these pathways is presented in Supplementary Table S2. We focused on those pathways related to cancer biology, cellular processes, renal disease and xenobiotic metabolism, and summaries of selected pathways for *Trp53(+/+)*, *Trp53(+/-),* and *Trp53(-/-)* kidneys are shown in Figure 4 (Supplementary Table S3). Overall, pathways related to immune response, epithelial-to-mesenchymal (EMT), transcription of hypoxia-inducible factor 1 (Hif-1) targets, renal injury, cell cycle, secretion of xenobiotics, and signalling processes in cancer were significant for *Trp53(+/+)*, *Trp53(+/-),* and *Trp53(-/-)* kidneys.

Gene lists for AAI-exposed *Trp53(+/+)*, *Trp53(+/-),* and *Trp53(-/-)* kidneys were also investigated manually. The top ten upregulated and downregulated genes were noted in the individual three gene lists (data not shown). Out of the top ten upregulated genes for each genotype, two genes in particular emerged for most of the genotypes; these were lipocalin 2 (*Lcn2*; fold change of 77.6 for *Trp53(+/+)*, 8.8 for *Trp53(+/-)* and 68.9 for *Trp53(-/-)*); and *Cdkn1a* (fold change of 16 for *Trp53(+/+)*, 8.4 for *Trp53(+/-)* and 9.6 for *Trp53(-/-)*). However, *Cdkn1a* was not among the top ten upregulated genes in *Trp53(-/-)* kidneys. *Slc* (i.e., *Slco1a1*, *Slc22a28*, *Slc22a30*, *Slc7a13*) were among the top ten downregulated genes for most of the genotypes.

### 2.4. Pathway Comparison for AAI-Exposed Trp53(+/+), Trp53(+/-), and Trp53(-/-) Kidneys

**Figure 5:**
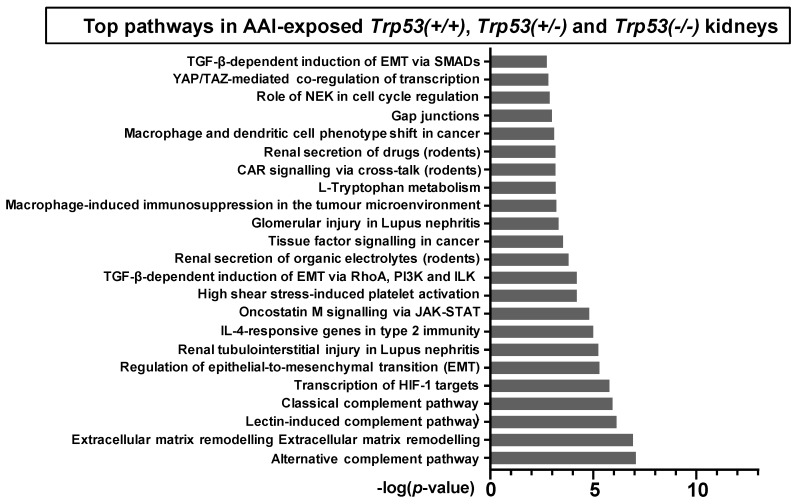
Top 23 pathways for genes in common for AAI-exposed *Trp53(+/+)*, *Trp53(+/-),* and *Trp53(-/-)* kidneys. The significance of the pathways are shown by the -log(*p*-value). Relevant pathways were selected out of top 50 pathways (*p* < 0.05). A brief pathway description, statistical significance (*p*-value and FDR), and the number of genes found within the pathway, including a list of these, are shown in Table S4. Analysis was carried out with MetaCore™.

**Figure 6:**
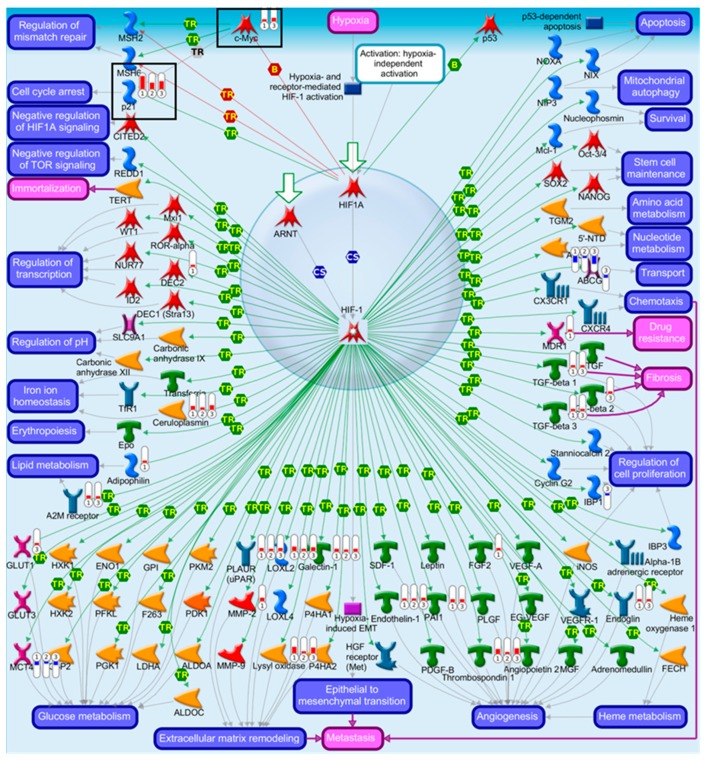
Map of transcription of HIF-1 targets. Significantly altered (*p* < 0.05; fold change ± 2) genes for AAI-exposed *Trp53(+/+)*, *Trp53(+/-),* and *Trp53(-/-)* mouse kidneys were compared in MetaCore™. The enriched pathway (Ninth out of top 50; *p* < 0.05) shows upregulated (thermometer-like symbols in red) and downregulated (thermometer-like symbols in blue) genes. Numbers indicate genotype: ① *Trp53(+/+)*, ② *Trp53(+/-),* and ③ *Trp53(-/-)*. Black boxes indicate genes of interest. For detailed legend see Figure S22. Abbreviation: HIF-1: hypoxia-inducible factor 1.

**Figure 7:**
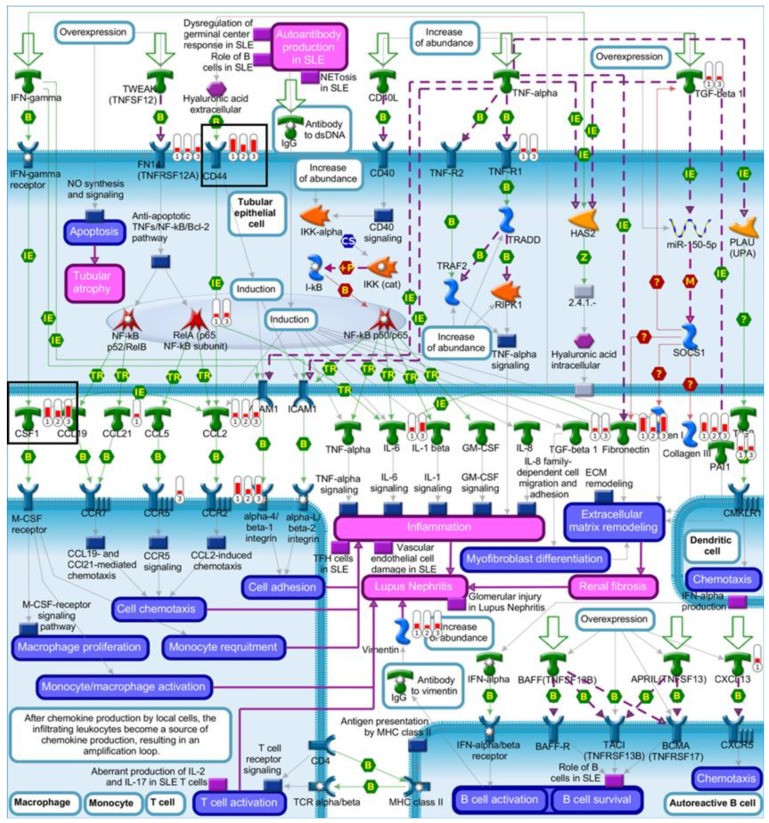
Map of renal tubulointerstitial injury in Lupus nephritis. Significantly altered (*p* < 0.05; fold change ± 2) genes for AAI-exposed *Trp53(+/+)*, *Trp53(+/-),* and *Trp53(-/-)* mouse kidneys were compared in MetaCore™. The enriched pathway (Twelfth out of top 50; *p* < 0.05) shows upregulated (thermometer-like symbols in red) genes. Numbers indicate genotype: ① *Trp53(+/+)*, ② *Trp53(+/-),* and ③ *Trp53(-/-)*. Black boxes indicate genes of interest. For detailed legend see Figure S22.

**Figure 8:**
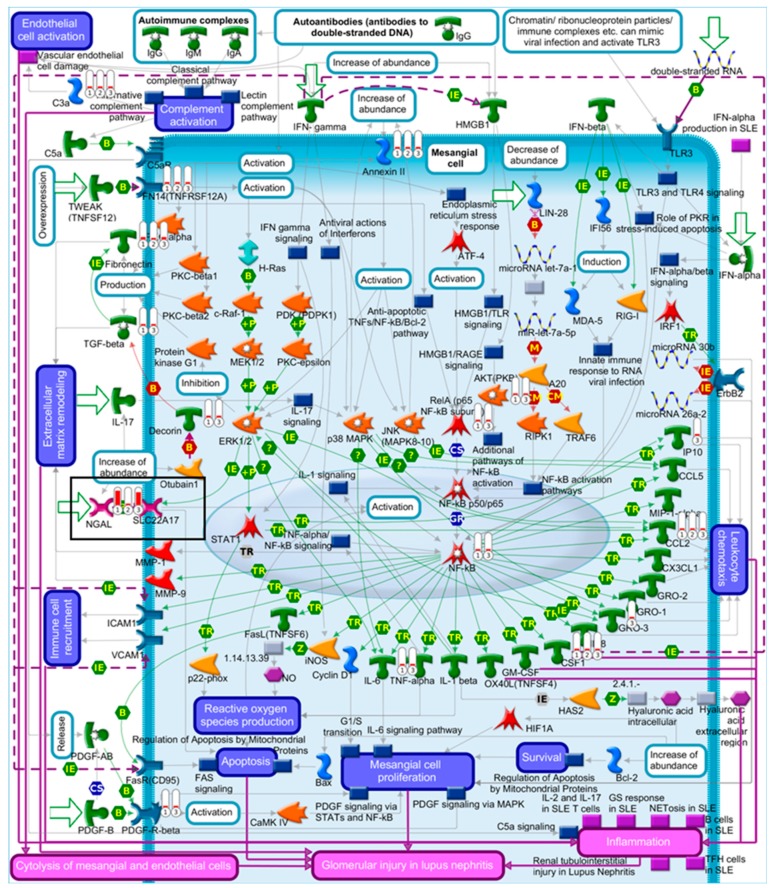
Map of glomerular injury in Lupus nephritis. Significantly altered (*p* < 0.05; fold change ± 2) genes for AAI-exposed *Trp53(+/+)*, *Trp53(+/-),* and *Trp53(-/-)* mouse kidneys were compared in MetaCore™. The enriched pathway (Twenty-ninth out of top 50; *p* < 0.05) shows upregulated (thermometer-like symbols in red) genes. Numbers indicate genotype: ① *Trp53(+/+)*, ② *Trp53(+/-),* and ③ *Trp53(-/-)*. Black boxes indicate genes of interest. For detailed legend see Figure S22.

**Figure 9:**
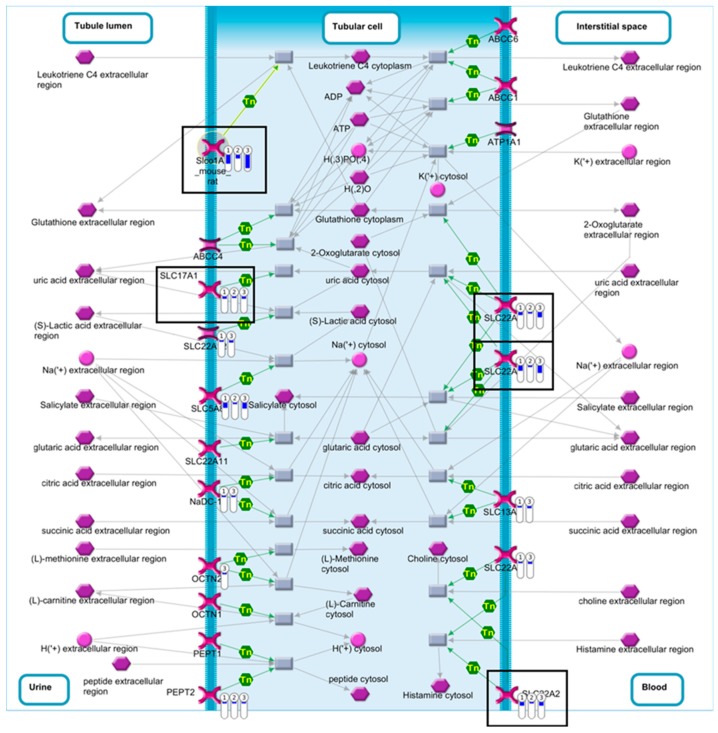
Map of renal secretion of organic electrolytes in rodents. Significantly altered (*p* < 0.05; fold change ± 2) genes for AAI-exposed *Trp53(+/+)*, *Trp53(+/-),* and *Trp53(-/-)* mouse kidneys were compared in MetaCore™. The enriched pathway (Twenty-fourth out of top 50; *p* < 0.05) shows downregulated (thermometer-like symbols in blue) genes. Numbers indicate genotype: ① *Trp53(+/+)*, ② *Trp53(+/-),* and ③ *Trp53(-/-)*. Black boxes indicate genes of interest. For detailed legend see Figure S22.

To investigate the *Trp53*-independent effects of AAI on gene expression, genes in common (318 genes) for AAI-exposed *Trp53(+/+)*, *Trp53(+/-),* and *Trp53(-/-)* kidneys were explored through pathway maps in MetaCore™. A summary of the obtained pathways (selected out of top 50, Supplementary Table S2; *p* < 0.05) for *Trp53(+/+)*, *Trp53(+/-),* and *Trp53(-/-)* kidneys is shown in Figure 5 (Supplementary Table S4). This analysis reflects the theme of pathways discussed above. Maps for selected pathways are shown in Figure 6, Figure 7, Figure 8 and Figure 9. A summary of these pathway maps is also shown in Table 1. The remainder of the pathway maps are presented in Figures S2–S20. As shown in Figure 6, *Cdkn1a* (i.e., p21) was upregulated in *Trp53(+/+)*, *Trp53(+/-),* and *Trp53(-/-)* kidneys, with the highest levels in *Trp53(+/+)* kidneys. Moreover, the c-*Myc* proto-oncogene was upregulated, with highest levels (fold change of 3.8) found in *Trp53(-/-)* kidneys (Figure 6). Pathways associated with injury of tubulointerstitial cells and glomeruli in Lupus nephritis, an autoimmune disease [32], were significant in *Trp53(+/+)*, *Trp53(+/-),* and *Trp53(-/-)* kidneys (Figure 7 and Figure 8). As shown in Figure 8, *Ngal* (also known as *Lcn2*) was upregulated in *Trp53(+/+)*, *Trp53(+/-),* and *Trp53(-/-)* kidneys. Furthermore, the expression of *Slc* organic anion and cation transporters (e.g., *Slc22*) [33] was downregulated in *Trp53(+/+)*, *Trp53(+/-),* and *Trp53(-/-)* kidneys following AAI treatment (Figure 9 and Supplementary Figure S2).

To investigate the *Trp53* genotype-dependent effects of AAI on gene expression, unique genes for AAI-exposed *Trp53(+/+)* (155 genes), *Trp53(+/-)* (4 genes), and *Trp53(-/-)* (352 genes) kidneys were individually explored through pathway maps in MetaCore™. Summaries of the obtained pathways for *Trp53(+/+)*, *Trp53(+/-),* and *Trp53(-/-)* kidneys are shown in Table 2. The top fifty pathways (*p* < 0.05) were investigated for the three genotypes (Supplementary Table S2). However, only four unique genes were found for *Trp53(+/-)* kidneys, thus a total of four pathway maps were obtained. The four genes modulated in exposed *Trp53(+/-)* kidneys were specifically glioma pathogenesis-related protein (*Glipr*), glutamate receptor 3 (*GluR3*), iron-regulated transporter 1 (*Irt1*), and *Ras*-related protein 2b (*Rap-2b*).

A total of 17 pathways were selected for *Trp53(+/+)* kidneys. Overall, unique genes for this genotype were involved in pathways related to the immune response, cellular metabolism, inflammation, apoptosis, stress response, transcription of Hif-1 targets, and regulation of EMT. One out of four genes for *Trp53(+/-)* kidneys was mapped to significant pathways. More precisely, the *Rap-2b* gene belonging to the *Ras* family of oncogenes [34]. A total of 22 pathways were selected for *Trp53(-/-)* kidneys. These pathways mapped to a wide range of cellular processes, including the immune response, cell cycle regulation, proliferation, metabolism, DNA replication and repair, and antiapoptotic responses.

## 3. Discussion

Microarrays are a powerful tool to examine whole-genome gene expression levels in a fast, simple, and high-throughput manner [35]. Given the exploratory approach of the present study, the Clariom™ S Assay array was used as a transcriptomics platform. In fact, it covers well-annotated genes (> 22,100) and it was also the most cost-effective array.

Previous work on kidneys isolated from *TP53(+/+)* Hupki mice demonstrated that AAI significantly alters gene expression [29]. In the present study, AAI treatment modulated gene expression in *Trp53(+/+)*, *Trp53(+/-),* and *Trp53(-/-)* kidneys. The number of genes modulated by AAI was higher in *Trp53(-/-)* kidneys (i.e., 1365 genes) in comparison to both *Trp53(+/+)* (i.e., 1180 genes) and *Trp53(+/-)* (i.e., 342 genes) kidneys. Intersections between genotypes demonstrated that *Trp53(+/+)* and *Trp53(+/-)* kidneys; and *Trp53(+/-)* and *Trp53(-/-)* kidneys share similarities in terms of AAI-induced gene expression. Differences between genotypes demonstrated that a higher number of AAI-modulated genes differ between *Trp53(+/+)* and both *Trp53(+/-)* and *Trp53(-/-)* kidneys. The number of genes modulated by AAI in *Trp53(+/-)* kidneys was low; and the fold change in gene expression was lower in AAI-exposed *Trp53(+/-)* kidneys relative to both AAI-exposed *Trp53(+/+)* and *Trp53(-/-)* kidneys. Overall, these findings indicated that AA-induced gene expression profiles are *Trp53* genotype-dependent. PCA analysis and hierarchical clustering further confirmed these findings. Hierarchical clustering demonstrated that control *Trp53(+/+)* kidneys separate from *Trp53(+/-)* and *Trp53(-/-)* kidneys, indicating that their biological differences impact on gene expression. The separation between *Trp53(+/+)*, *Trp53(+/-),* and *Trp53(-/-)* kidneys was less pronounced in the AAI-treated group. However, most of the AAI-exposed *Trp53(-/-)* kidneys clustered together.

Pathway analysis on an individual and comparative basis for AAI-exposed *Trp53(+/+)*, *Trp53(+/-),* and *Trp53(-/-)* kidneys demonstrated that AAI affects certain biological processes. These can be broadly subdivided into the following categories: transcription, renal injury, secretion of xenobiotics, cell cycle, immune response, cell adhesion and development, tissue damage, cancer-related processes, and metabolism. Some of the observed genes (e.g., c-*Myc*) and pathways were also found in previous work on AAI-treated *TP53(+/+)* Hupki mice [29].

p21 (encoded by *Cdkn1a*) is a mediator of cell cycle arrest [36]. Pathway analysis demonstrated that *Cdkn1a* is upregulated in *Trp53(+/+)*, *Trp53(+/-),* and *Trp53(-/-)* kidneys. This is in line with previous findings showing upregulation of *Cdkn1a* in kidneys from AAI-treated *TP53(+/+)* Hupki mice [29]. Previous in vitro work in human proximal tubular epithelial HK-2 and hepatoma HepG2 cells demonstrated that AAI induces cell cycle arrest via p21 [37,38]. Moreover, the development of fibrosis in kidney proximal tubules is p21-dependent [39]. In the present study, the expression of *Cdkn1a* was highest in *Trp53(+/+)* kidneys. This was expected since *Trp53(+/+)* mice have the highest allelic dosage of p53 and p21 is a major target of p53 [36]. The finding could also indicate that *Trp53(+/+)* cells have the capacity to overcome AAI-induced damage by inducing cell cycle arrest.

The proto-oncogene c-*Myc* was also upregulated within the Hif-pathway, particularly in *Trp53(+/+)* and *Trp53(-/-)* kidneys. Previous work on AAI-exposed HCT116 cells of differing *TP53* genotypes and in *TP53(+/+)* Hupki mice demonstrated c-*MYC* upregulation [28,29]. Furthermore, c-*MYC* over-expression is typical of urothelial cancers [40,41,42].

Pathways related to renal damage were modulated by AAI in *Trp53(+/+)*, *Trp53(+/-),* and *Trp53(-/-)* mice. The obtained pathways were associated with Lupus nephritis, an autoimmune disease characterised by renal inflammation and glomerular damage [32]. However, a number of genes within these pathways are also relevant to AAI-induced renal injury. For example, *Ngal* (i.e., *Lcn2*) was significantly upregulated in *Trp53(+/+)*, *Trp53(+/-),* and *Trp53(-/-)* kidneys. NGAL is a protein that binds to iron and it is over-expressed in renal disease [43,44]. Previous work demonstrated that *Ngal* plays a role in nephritis by promoting inflammation and apoptosis [43]. *NGAL* is also over-expressed in human cancers [45]. In terms of AAI treatment, previous in vivo work demonstrated that *Ngal* can be used as a biomarker of exposure [46]. Moreover, *LCN2* was upregulated in AAI-exposed *TP53(+/+)* and *TP53(-/-)* HCT116 cells [28]. In respect to AAI-induced damage, it is noteworthy that AAI-DNA adducts formed in *Trp53(+/+)*, *Trp53(+/-),* and *Trp53(-/-)* kidneys at a similar level and *Trp53* status did not impact on AAI bioactivation [31]. In fact, pathways related to AAI bioactivation and AAI-induced DNA damage were not observed in the present study.

Inflammatory cells are a feature of AAN [47] and macrophages play an important role in AAN pathogenesis [48]. Cluster of differentiation 44 (*Cd44*) and colony-stimulating factor 1 (*Csf-1*) were upregulated in *Trp53(+/+)*, *Trp53(+/-),* and *Trp53(-/-)* kidneys. CD44 is a glycoprotein that is expressed on immune cells, whereas CSF-1 is a growth factor for macrophages; and both of these genes are upregulated in nephritis [49,50,51]. Moreover, a number of immune response pathways (e.g., alternative complement pathway) were significantly altered in *Trp53(+/+)*, *Trp53(+/-),* and *Trp53(-/-)* kidneys. Thus, indicating that AAI modulates the immune response.

Transporters of the SLC22 family include organic cation transporters (OCTs) and OATs, whereas transporters of the SLCO family consist of organic anion transporting polypeptides (OATPs) [33,52]. The main function of OCTs, OATs, and OATPs is to absorb, excrete, and distribute xenobiotics in tissues (e.g., kidneys) [33,52]. The following *Slc22* and *Slco* genes were downregulated in *Trp53(+/+)*, *Trp53(+/-),* and *Trp53(-/-)* kidneys: *Slc22a2* (i.e., *Oct2*), *Slc22a6* (i.e., *Oat1*), *Slc22a7* (i.e., *Oat2*), *Slc22a8* (i.e., *Oat3*), and *Slco1a1* (i.e., *Oatp1a1*) [33,52]. Another OAT belonging to the SLC17 family (i.e., *Slc17a1*) [53] was also downregulated in the present study. OATs are located in the basolateral membrane of proximal tubules and transport drugs from the bloodstream into proximal tubular cells [15]. Previous in vitro and in vivo work demonstrated that OAT1, OAT2, and OAT3 modulate AAI uptake in proximal tubular cells, where AAI-induced damage occurs [54,55,56,57]. Interestingly, a study on AA-treated (10 and 20 mg/kg bw) rats showed a significant decrease in *Oat1*, *Oat3,* and *Oct2* levels in kidney [58]. The decrease in *Oat1* and *Oat3* expression could be explained by the fact that AAI decreased the uptake of OAT1- and OAT3-specific substrates (i.e., *p*-aminohippurate and estrone sulfate, respectively) in human epithelial kidney HEK-293 cells [55]. A reduction in *Oct2* expression could indicate that this transporter is unable to excrete AAI from proximal tubular cells [59]. Overall, these findings indicated that AAI affects the transport of ions within the kidney and damage of ion transporters potentially contributes to AAI-induced nephrotoxicity.

Transcriptomics analysis on *Trp53(+/+)*, *Trp53(+/-),* and *Trp53(-/-)* kidneys also indicated gene expression changes in pathways related to cell adhesion (e.g., regulation of EMT) and development (e.g., TGF-β-dependent induction of EMT). EMT is a process whereby biochemical changes allow for epithelial cells to acquire mesenchymal features, which include migration and many others [60]. EMT is a mechanism by which renal tubular cells induce the formation of fibrosis [61]. AAN is characterised by fibrosis [62], thus it could be postulated that AAI contributes to this phenomenon by inducing EMT. In fact, previous studies showed that AAI-induced upregulation of transforming growth factor β (TGF-β), which acts on EMT pathways, contributes to the formation of renal fibrosis in AAN [63,64,65].

It was of importance to explore AAI-induced gene expression changes unique to *Trp53(+/+)*, *Trp53(+/-),* and *Trp53(-/-)* kidneys.

Pathways related to the immune response, transcription of Hif-1 targets and regulation of EMT were significant for *Trp53(+/+)* kidneys. Two pathways related to apoptosis were significantly altered in *Trp53(+/+)* kidneys, namely the ceramides and lymphotoxin-β receptor (L-βR) signalling pathways. The former pathway induces apoptosis; whereas the latter pathway plays a role in lymphoid tissue development, chemokine release, apoptosis, and NF-κB (nuclear factor kappa-light-chain-enhancer of activated B cells) activation [66,67]. L-βR also mediates apoptosis in various cancer cells [68]. A number of genes were upregulated in both pathways in *Trp53(+/+)* kidneys. For example, the apoptotic protease activating factor 1 (*Apaf-1*) and transcription factor *c-Jun* were both upregulated in such tissues. The former gene activates a caspase cascade as part of the L-βR pathway, whereas the latter gene is a member of the ceramides pathway [67,69]. Previous in vitro work demonstrated that AA induces the expression of TGF-β1 by activating the apoptotic c-Jun N-terminal kinase (JNK) pathway, of which *APAF-1* and *c-Jun* are key players [70]. Moreover, previous in vivo work showed that injury in AA-exposed *Trp53(+/+)* kidneys is driven by an apoptotic mechanism [71]. Several apoptosis-related genes were also modulated by AAI in kidneys of *TP53(+/+)* Hupki mice [29].

Only four genes (i.e., *Glipr*, *GluR3*, *Irt1*, *Rap-2b*) were unique for *Trp53(+/-)* kidneys, indicating that these tissues share most gene expression changes with both *Trp53(+/+)* and *Trp53(-/-)* tissues. Expression of *Rap-2b* was of significance in pathway analysis. As forementioned, *Rap-2b* is classified as an oncogene and it is over-expressed in cancers [34,72]. Previous work demonstrated that *Rap-2b* is a target of p53 and it counteracts p53-mediated apoptosis [72]. Moreover, *Rap-2b* can affect cytoskeleton reorganisation and cell migration [73,74]. Given the role of *Rap-2b* in transformation, it may be that upregulation of this gene contributes to AAI-induced carcinogenesis.

The highest number of unique genes was detected for *Trp53(-/-)* kidneys. Significant pathways were related to the cell cycle, antiapoptotic responses, cytoskeleton remodelling, immune response, DNA damage, metabolism, transport, cellular signalling, and transcription. Overall, indicating that AAI modulates transformation processes in *Trp53(-/-)* kidneys.

One of the genes that was upregulated as part of cell cycle-related processes in *Trp53(-/-)* kidneys was *Cdk2*. This is a regulator of the cell cycle, particularly in the G_1_-S transition. Its deregulation indicates a dysfunction in cell cycle regulation or DNA repair [75,76]. Previous in vitro studies demonstrated that AAI induces cell cycle arrest in the G_2_-M transition [37,77]. However, work on rodents showed that AAI-induced proliferation of urothelial cells is a consequence of cell cycle progression, specifically through an increase in Cdk4-cyclin D_1_ and Cdk2-cyclin E [78]. Cyclin E was also over-expressed in human urothelial cancer [79], thus indicating that these cell cycle members can contribute to malignancy.

DNA damage and repair pathways were also significantly altered in *Trp53(-/-)* kidneys. Upregulated genes in such pathways included the tumour suppressors *Brca1* (breast cancer susceptibility gene 1) and *Brca2*. Not only do these genes confer a susceptibility to breast and ovarian cancers, but they also play an important role in maintaining genomic stability by interacting with numerous regulators [80]. For example, BRCA1 and BRCA2 respond to DNA damage (e.g., double-strand breaks) by interacting with the repair protein RAD-51 [80,81]. Previous in vitro studies demonstrated the formation of double-strand breaks following AA exposure [82]. Our recent work also showed that expression of H2ax, a marker for double-strand breaks, is highest in AAI-exposed *Trp53(-/-)* kidneys [31]. Whole-exome sequencing revealed *BRCA2* mutations in urothelial cancers associated with AA exposure [83,84]. Furthermore, previous work showed that *BRCA1* can interact with DNA repair and cell cycle genes modulated by AAI [63]. It may be that *Brca* genes drive DNA repair pathways in response to AAI treatment. However, deregulation of *Brca* genes may also indicate a defect in DNA repair and malignant transformation.

Survival-related pathways were altered in *Trp53(-/-)* kidneys. The following members of the antiapoptotic Tnf/Nf-κb/Iap pathway were upregulated: *RelA* (i.e., subunit for *Nf-κb*), *Nf-κb*, *c-Iap2,* and *Survivin*. Inhibitor of apoptosis (IAP) proteins can inhibit caspases [85]. Moreover, their indirect interaction with TNF receptors can activate the pro-survival NF-κB pathway [85]. Previous in vivo work showed that *Nf-κb* and members of its pathway are upregulated in AAI-exposed kidneys [29]. NF-κB also plays a role in the inflammatory response associated with AAN [63]. Overall, these findings may indicate that a *Trp53(-/-)* genotype confers a survival advantage to AAI-exposed renal cells and/or an inflammatory response is initiated in injured *Trp53(-/-)* kidneys.

Renal fibrosis and chronic kidney disease are associated with changes in fatty acid oxidation (FAO), cytoskeletal remodelling, EMT, and inflammation [86,87]. A transition from acute to chronic inflammation is associated with a switch from glycolysis to FAO [88]. Glucose transporters (GLUT), which mediate glycolysis, are located in proximal tubular cells [89]. Previous in vivo work associated AA treatment with changes in lipid metabolism and FAO [58,90]. In the present study, *Glut1* was upregulated in *Trp53(-/-)* kidneys as part of the Sirtuin 6 pathway. Sirtuin proteins regulate both glucose and lipid metabolism; and act on switching the two forms of metabolism [88,91]. Given that p53 plays a role in glucose metabolism by downregulating the expression of glucose transporters (e.g., *GLUT1*, *GLUT4*) [92], it may be that a *Trp53(-/-)* genotype confers a deregulation in *Glut1* expression and drives glycolysis. This phenomenon may potentially drive an acute inflammatory response in *Trp53(-/-)* kidneys.

## 4. Materials and Methods

### 4.1. Carcinogen

Aristolochic acid I (CAS Number: 10190-99-5; AAI; as sodium salt) was isolated as previously reported [93].

### 4.2. Maintenance of Trp53(+/+), Trp53(+/-), and Trp53(-/-) Mice

*Trp53(+/+)*, *Trp53(+/-),* and *Trp53(-/-)* C57BL/6 mice were generated as previously reported [4] and kindly provided by Mirjam Luijten from the National Institute for Public Health and the Environment (RIVM), Bilthoven, The Netherlands [94,95]. *Trp53(+/-)* and *Trp53(-/-)* mice carry a neomycin cassette that replaces exons 2 and 6 of the *Trp53* gene, thus eliminating the synthesis of p53 protein [96,97]. *Trp53(-/-)* mice are viable and their initial development is normal; however, they develop tumours (mostly lymphomas) at 3–6 months of age [96,97]. *Trp53(+/-)* mice develop sarcomas at approximately 18 months of age [96,98]. More information about the *Trp53^tm1Tyj^* mouse strain can be found at www.jax.org/strain/002101. All animal experiments were carried out at King’s College London under licence (Reference number X24D82DFF) in accordance with the Animal (Scientific Procedures) Act (1986), as amended by EU Directive 2010/63/EU, and with local ethical approval. Mice were bred at the Biological Services Unit at King’s College London by a *Trp53(+/-)* × *Trp53(+/-)* strategy to maintain the colony and produce *Trp53(+/+)*, *Trp53(+/-),* and *Trp53(-/-)* mice for experiments. All mice were maintained under controlled pathogen-free conditions with food and water ad libitum and 12 h light/dark cycle.

*Trp53* genotype was determined in mouse pups by PCR prior to experiments. Ear biopsies were taken from mice at 2–3 weeks of age and DNA was extracted as previously described [99]. PCR was performed according to the manufacturer’s instructions by using a 2X REDTaq ReadyMix PCR Reaction Mix with MgCl_2_ (Sigma-Aldrich, St. Louis, MO, USA). Primers and PCR reaction conditions for an Eppendorf Mastercycler are described in Supplementary Table S5. PCR products were run on a 2% UltraPure agarose gel (Supplementary Figure S21). DNA from *Trp53(+/+)* and *Trp53(-/-)* mice resulted in one band of 321 and 110 bp, respectively; whereas DNA from *Trp53(+/-)* mice resulted in two bands, one at 321 bp and the other at 110 bp.

### 4.3. Treatment of Trp53(+/+), Trp53(+/-), and Trp53(-/-) Mice with AAI

*Trp53(+/+)*, *Trp53(+/-),* and *Trp53(-/-)* male mice (9–11 weeks of age; *n* = 5/group) were treated with 3.5 mg/kg bw AAI by intraperitoneal (i.p.) injection daily for six days (Figure 1b) on the basis of a previously established protocol to study experimental AAN [100]. The dose to inject per mouse was determined by weighing the mice one day in advance or on the first day of the experimental protocol. Control mice (*n* = 5/group) were injected with water only. Mice were euthanised 24 h after the last treatment using a rising concentration of CO_2_; and kidneys were collected, snap frozen in liquid nitrogen, and stored at ‒80 °C for further analysis.

### 4.4. Microarray

Total RNA was isolated by a modified method based on both TRIzol^®^ (Thermo Fisher Scientific, Waltham, MA, USA) and RNeasy Mini Kit (QIAGEN, Venlo, The Netherlands) protocols. A portion of tissue (15–35 mg) was placed in a tube containing a steel bead and 1 ml of TRIzol^®^. The tissue was homogenised twice with a TissueLyser II at 25 Hz for 2 min and it was placed at room temperature for 5 min. Following the addition of 200 µL of chloroform, it was centrifuged at 4 °C at 13,000 rpm (5424R, Eppendorf™, Hamburg, Germany) for 20 min. The top layer was transferred to a tube and mixed with 350 µL of 70% ethanol. The sample was transferred to a RNeasy Mini Spin column and subsequent RNA isolation steps were performed according to the manufacturer’s instructions. On-column DNase digestion with an RNase-Free DNase Set (QIAGEN) was also performed according to the manufacturer’s instructions. The concentration and purity (260/280 ratio of 2) of the RNA was measured with a NanoDrop™ 2000 Spectrophotometer (Thermo Fisher Scientific, Waltham, MA, USA). An aliquot of RNA was used to measure the integrity of the RNA as described below. Total RNA was stored at ‒80 °C before performing microarray analysis.

The integrity of the total RNA was determined with the Agilent RNA 6000 Nano Kit (Agilent Technologies, Santa Clara, CA, USA) according to the manufacturer’s instructions. Total RNA was diluted to a concentration in the range of 25‒500 ng/µL with RNase-free water. The prepared Nano chip was vortexed with an IKA vortex mixer (Applied Biosystems™, Waltham, MA, USA) for 1 min at 2400 rpm and analysed with the Agilent 2100 Bioanalyzer System. The RNA samples selected for subsequent microarray analysis had the following properties: a 260/280 purity ratio of 2, a concentration of > 200 ng/µL, and an RNA integrity number (RIN) of ≥ 7.

Microarray analysis was conducted at the Genomics Centre at King’s College London and performed on total RNA isolated from *Trp53(+/+)*, *Trp53(+/-),* and *Trp53(-/-)* mouse kidneys exposed to water (control) or AAI for six days (*n* = 5/group). Total RNA (50 ng) was converted and amplified into cDNA with the Ovation^®^ Pico WTA System V2 Kit (NuGEN, Redwood City, CA, USA) before hybridisation onto an array. A GeneChip™ Poly-A RNA Control (Thermo Fisher Scientific) was used as an amplification control. In brief, the aforementioned kit utilises single primer isothermal amplification (SPIA^®^) technology to generate cDNA according to the manufacturer’s instructions. The SPIA^®^ cDNA was subjected to quality control (QC) with the Agilent RNA 6000 Nano Kit and quantified with a NanoDrop™ 2000 Spectrophotometer. The SPIA^®^ cDNA was fragmented and biotin-labelled with the Encore^®^ Biotin Module (NuGen) according to the manufacturer’s instructions. To assess fragmentation size (< 200 nucleotides), the resulting cDNA was subjected to a further round of QC with the Agilent RNA 6000 Nano Kit. Note that cDNA synthesis was performed on two separate occasions, one with a batch of 14 samples (i.e., batch #1) and another with a batch of 16 samples (i.e., batch #2). Hybridisation cocktails using the fragmented and biotin-labelled cDNA were prepared according to NuGen’s recommendations for mouse Clariom™ S Assay (Thermo Fisher Scientific) arrays. The Clariom™ S Assay allows to investigate the gene expression levels from > 20,000 well-annotated genes. Hybridisation took place at 45 °C for 16‒20 h at 60 rpm in a GeneChip™ Hybridization Oven 645 (Thermo Fisher Scientific). The arrays were washed and stained on a GeneChip™ Fluidics Station 450 (Thermo Fisher Scientific) by using a recommended fluidics protocol (FS450_0007, Affymetrix, Santa Clara, CA, USA). The arrays were scanned with the GeneChip™ Scanner 3000 7G (Thermo Fisher Scientific).

### 4.5. Microarray Data Analysis

The data files were QC checked by using the Transcriptome Analysis Console (TAC) software (Thermo Fisher Scientific). This was performed by using standard metrics and guidelines for the microarray system. The data was normalised using the Robust Multi-array Average (RMA) sketch algorithm. The RMA normalised data were analysed visually with the Qlucore Omics Explorer software. Gene lists were created in Qlucore Omics Explorer according to the biological question taken into consideration and by taking the following parameters into account: *p*-value (*p*) < 0.05 and fold change cut-off ± 2. Statistical analyses in Qlucore Omics Explorer were based on an analysis of variance (ANOVA) test. The false discovery rate (FDR) was kept to approximately 15% and batch effects were also eliminated. Pathway analysis was carried out with the MetaCore™ software (Clarivate Analytics, Philadelphia, PA, USA), particularly using the “Enrichment analysis in Pathway Maps” and “Compare Experiments Workflow” tools. Pathway significance was set to *p* < 0.05. The MetaCore™ pathway analysis software was utilised as it is manually curated; and it provides > 1.7 million molecular interactions, > 1600 pathway maps, and > 230,000 gene-disease associations. Further information on pathway maps obtained from MetaCore™ is shown in Supplementary Figure S22.

The gene expression data discussed in this publication have been deposited in and are accessible through the accession number GSE136276.

## 5. Conclusions

Microarray analysis on AAI-exposed *Trp53(+/+)*, *Trp53(+/-),* and *Trp53(-/-)* kidneys revealed treatment-dependent changes in gene expression and several biological pathways. For example, the impact of AAI treatment on the immune response, cell cycle arrest and ion transport within the kidney were shown by changes in the expression of *Cdkn1a*, c-*Myc*, *Ngal,* and *Slc* genes. Pathways related to apoptosis were significantly modulated in *Trp53(+/+)* kidneys, potentially indicating a protective effect in response to AAI treatment. The significant modulation of the *Rap-2b* gene in *Trp53(+/-)* kidneys suggests a transformative mechanism of AAI. A number of genes (e.g., *Cdk2*, *Brca1/2*, *Nf-κb*, *Glut*) involved in cell cycle, DNA damage or repair, and inflammation were modulated in *Trp53(-/-)* kidneys. This indicated the potential ways in which renal injury is induced or driven in such tissue. Overall, the findings presented in this study provided novel insights into the ways in which p53 impacts on AAI-related nephrotoxicity and carcinogenesis in vivo.

## Figures and Tables

**Table 1:** Relevant pathways modulated in AAI-exposed *Trp53(+/+)*, *Trp53(+/-,)* and *Trp53(-/-)* kidneys. A brief pathway description, statistical significance (*p*-value and FDR), and the number of genes found within the pathway, including a list of these, are shown. Analysis was carried out with MetaCore™. Abbreviation: FDR: False discovery rate.

Pathway	*p*-Value	FDR	No. Genes	List of Genes from MetaCore™
Transcription of HIF-1 targets	1.67 × 10^−6^	1.43 × 10^−4^	10	*Galectin-1, PLAUR (uPAR), Ceruloplasmin, Lysyl oxidase, p21, MCT4, Endothelin-1, Thrombospondin 1, LOXL2, AK3*
Renal tubulointerstitial injury in Lupus nephritis	5.80 × 10^−6^	3.74 × 10^−4^	8	*CSF1, CD44, CCL2, Fibronectin, Vimentin, FN14(TNFRSF12A), CCR2, Collagen III*
Glomerular injury in Lupus nephritis	4.88 × 10^−4^	1.24 × 10^−2^	7	*CSF1, CCL2, Fibronectin, C3a, NGAL, Annexin II, FN14(TNFRSF12A)*
Renal secretion of organic electrolytes (rodents)	1.62 × 10^−4^	5.22 × 10^−3^	7	*SLC17A1, SLC22A2, SLC22A8, Slco1a1, SLC5A8, SLC22A6, PEPT2*

**Table 2:** Top 17, 3, and 22 pathways for genes unique to AAI-exposed *Trp53(+/+)*, *Trp53(+/-),* and *Trp53(-/-)* kidneys. Relevant pathways were selected out of top 50 (or 4 for *Trp53(+/-)* kidneys) pathways (*p* < 0.05). The rank indicates the position of the pathway within the top 50 (or 4). A brief pathway description, statistical significance (*p*-value and FDR) and the number of genes found within the pathway, including a list of these, are shown. Analysis was carried out with MetaCore™. Abbreviation: FDR: False discovery rate.

ID	Rank	Pathway	*p*-Value	FDR	No. Genes	List of Genes from MetaCore™
***Trp53(+/+)***						
1	2	GTP metabolism	2.44 × 10^−6^	9.90 × 10^−4^	6	*GUCY1B1, GUCY1A3, GUCY1A2, Guanylate cyclase beta, Guanylate Cyclase 1, soluble, Guanylate cyclase alpha*
2	4	IL-5 signalling via PI3K, MAPK and NF-kB	1.82 × 10^−5^	3.69 × 10^−3^	6	*AP-1, c-Jun, Calpastatin, PI3K reg class IA (p85), MMP-2, Fc gamma RII alpha*
3	7	CCL2 signalling	4.35 × 10^−5^	5.05 × 10^−3^	5	*AP-1, c-Jun, ZO-1, PI3K reg class IA (p85), MMP-2*
4	15	Ceramides signalling pathway	1.96 × 10^−4^	1.03 × 10^−2^	4	*c-Jun, PI3K reg class IA (p85-alpha), PI3K reg class IA (p85), Cathepsin D*
5	18	Lymphotoxin-β receptor signalling	2.38 × 10^−4^	1.07 × 10^−2^	4	*Apaf-1, c-Jun, CXCL13, CCL21*
6	21	TNF-R2 signalling pathways	3.11 × 10^−4^	1.20 × 10^−2^	4	*AP-1, c-Jun, PI3K reg class IA (p85), PI3K reg class IA*
7	23	PTMs in IL-17-induced CIKS-independent signalling pathways	3.39 × 10^−4^	1.20 × 10^−2^	4	*AP-1, c-Jun, PI3K reg class IA (p85), PI3K reg class IA*
8	24	TGF-β-dependent induction of epithelial-to-mesenchymal transition (EMT) via MAPK	3.69 × 10^−4^	1.25 × 10^−2^	4	*ITGB1, AP-1, c-Jun, MMP-2*
9	25	FGF2-dependent induction of EMT	3.89 × 10^−4^	1.26 × 10^−2^	3	*FGF2, AP-1, PI3K reg class IA (p85)*
10	26	PEDF signalling	4.33 × 10^−4^	1.35 × 10^−2^	4	*SOD2, Fra-2, PI3K reg class IA, NGF*
11	31	IL-4 signalling pathway	6.02 × 10^−4^	1.51 × 10^−2^	5	*AP-1, c-Jun, Fra-2, PI3K reg class IA (p85-alpha), c-Jun/Fra-2*
12	32	HSP60 and HSP70/TLR signalling pathway	6.29 × 10^−4^	1.51 × 10^−2^	4	*AP-1, c-Jun, CD14, HSP60*
13	34	Transcription of HIF-1 targets	6.31 × 10^−4^	1.51 × 10^−2^	5	*FGF2, MDR1, Adipophilin, MMP-2, DEC2*
14	37	TLR5, TLR7, TLR8 and TLR9 signalling pathways	7.72 × 10^−4^	1.70 × 10^−2^	4	*AP-1, c-Jun, PI3K reg class IA (p85), TLR8*
15	42	Adenosine A1 receptor signalling pathway	1.06 × 10^−3^	1.98 × 10^−2^	4	*SFK, PI3K reg class IA (p85), MMP-2, ADA*
16	45	IL-18 signalling	1.13 × 10^−3^	1.98 × 10^−2^	4	*AP-1, c-Jun, PI3K reg class IA (p85-alpha), PI3K reg class IA*
17	48	Regulation of EMT	1.20 × 10^−3^	1.98 × 10^−2^	4	*FGF2, c-Jun, ZO-1, MMP-2*
***Trp53(+/-)***						
1	1	RAP2B regulation pathway	6.07 × 10^−4^	2.43 × 10^−3^	1	*RAP-2B*
2	3	Regulation of cyclic AMP levels by ACM	3.90 × 10^−3^	4.08 × 10^−3^	1	*RAP-2B*
3	4	β-adrenergic receptor-induced regulation of ERK	4.08 × 10^−3^	4.08 × 10^−3^	1	*RAP-2B*
***Trp53(-/-)***						
1	1	The metaphase checkpoint	1.42 × 10^−7^	1.36 × 10^−4^	8	*INCENP, CDCA1, CDC20, Rod, CENP-F, MAD2a, Survivin, CENP-H*
2	2	Spindle assembly and chromosome separation	1.90 × 10^−5^	9.05 × 10^−3^	6	*KNSL1, Importin (karyopherin)-alpha, CDC20, TPX2, MAD2a, Importin (karyopherin)-beta*
3	4	dCTP/dUTP metabolism	2.87 × 10^−4^	6.85 × 10^−2^	7	*POLE1, Small RR subunit, RRM1, POLA2, Ribonucleotide reductase, RRM2, POLA1*
4	6	Transition and termination of DNA replication	8.36 × 10^−4^	1.10 × 10^−1^	4	*PCNA, Brca1, DNA ligase I, CDK2*
5	7	Anti-apoptotic TNFs/NF-kB/IAP pathway	1.13 × 10^−3^	1.10 × 10^−1^	4	*RelA (p65 NF-kB subunit), NF-kB, Survivin, c-IAP2*
6	8	Regulation of actin cytoskeleton nucleation and polymerization by Rho GTPases	1.15 × 10^−3^	1.10 × 10^−1^	5	*F-Actin cytoskeleton, FMNL1, mDIA2(DIAPH3), DRF, Actin cytoskeletal*
7	9	IFN-α/β signalling via PI3K and NF-kB pathways	1.22 × 10^−3^	1.10 × 10^−1^	7	*PCNA, b-Myb, RelA (p65 NF-kB subunit), NF-kB, p107, CDK2, ISG15*
8	10	dATP/dITP metabolism	1.22 × 10^−3^	1.10 × 10^−1^	7	*POLE1, Small RR subunit, RRM1, POLA2, Ribonucleotide reductase, RRM2, POLA1*
9	13	Nucleocytoplasmic transport of CDK/cyclins	1.63 × 10^−3^	1.15 × 10^−1^	3	*Importin (karyopherin)-alpha, CDK2, Karyopherin beta 1*
10	14	Role of BRCA1 and BRCA2 in DNA repair	1.69 × 10^−3^	1.15 × 10^−1^	4	*PCNA, Brca1, Rad51, Brca2*
11	16	ATM/ATR regulation of G1/S checkpoint	2.16 × 10^−3^	1.15 × 10^−1^	4	*PCNA, Brca1, NF-kB, CDK2*
12	17	Role of APC in cell cycle regulation	2.16 × 10^−3^	1.15 × 10^−1^	4	*CDC20, MAD2a, Emi1, CDK2*
13	18	Start of DNA replication in early S phase	2.16 × 10^−3^	1.15 × 10^−1^	4	*ASK (Dbf4), MCM4, CDC7, CDK2*
14	24	RAN regulation pathway	3.46 × 10^−3^	1.32 × 10^−1^	3	*RanBP1, Importin (karyopherin)-alpha, Importin (karyopherin)-beta*
15	25	RAC1 in cellular process	3.71 × 10^−3^	1.32 × 10^−1^	4	*F-Actin cytoskeleton, gp91-phox, Actin cytoskeletal, PARD6*
16	29	IL-9 signalling pathway	4.34 × 10^−3^	1.32 × 10^−1^	5	*IL-2R gamma chain, Scinderin, mTOR, Eotaxin, CCL7*
17	30	Macropinocytosis regulation by growth factors	4.65 × 10^−3^	1.32 × 10^−1^	5	*AMPK beta subunit, Leptin receptor, AMPK alpha subunit, PDE3B, Actin cytoskeletal*
18	31	Inhibition of telomerase activity and cellular senescence	4.72 × 10^−3^	1.32 × 10^−1^	3	*Brca1, p107, CDK2*
19	36	Sirtuin 6 regulation and functions	4.98 × 10^−3^	1.32 × 10^−1^	5	*AMPK beta subunit, AMPK alpha subunit, RelA (p65 NF-kB subunit), c-IAP2, GLUT1*
20	45	ChREBP regulation pathway	7.05 × 10^−3^	1.46 × 10^−1^	3	*AMPK beta subunit, AMPK alpha subunit, Acyl-CoA synthetase*
21	46	CDC42 in cellular processes	7.05 × 10^−3^	1.46 × 10^−1^	3	*F-Actin cytoskeleton, Actin cytoskeletal, PARD6*
22	50	Leptin signalling via PI3K-dependent pathway	8.76 × 10^−3^	1.64 × 10^−1^	4	*AMPK beta subunit, Leptin receptor, AMPK alpha subunit, PDE3B*

## Supplementary Materials

Supplementary materials can be found at https://www.mdpi.com/1422-0067/20/24/6155/s1.

## Abbreviations

AAI	aristolochic acid I
AAN	aristolochic acid nephropathy
BEN	Balkan endemic nephropathy
*BRCA*	breast cancer susceptibility gene
bw	body weight
CDK	cyclin-dependent kinase
CYP	cytochrome P450
EMT	epithelial-to-mesenchymal transition
FDR	false discovery rate
GLUT	glucose transporter
HIF-1	hypoxia-inducible factor 1
Hupki	human *TP53* knock-in
i.p.	intraperitoneal
NF-κB	nuclear factor kappa-light-chain-enhancer of activated B cells
NGAL or LCN2	neutrophil gelatinase-associated lipocalin
NQO	NAD(P)H:quinone oxidoreductase
OAT	organic anion transporter
*p*	*p*-value
p21^Cip1/Waf1^ or CDKN1A	cyclin-dependent kinase inhibitor 1a
p53	tumour protein p53
QC	quality control
SLC	solute carrier
*TP53*	tumour protein 53 gene (human)
*Trp53*	tumour protein 53 gene (mouse)
