# Supplementary material for: The Impact of p53 on Aristolochic Acid I-Induced Gene Expression In Vivo
Source: Int J Mol Sci. 2019 Dec 6;20(24):6155. doi: 10.3390/ijms20246155 (PMC6940885; doi:10.3390/ijms20246155)
Supplement: Supplementary file 1 [file ijms-20-06155-s001.zip › ijms-649433-supplementary-figures and tables/Supplementary_figures.pdf]

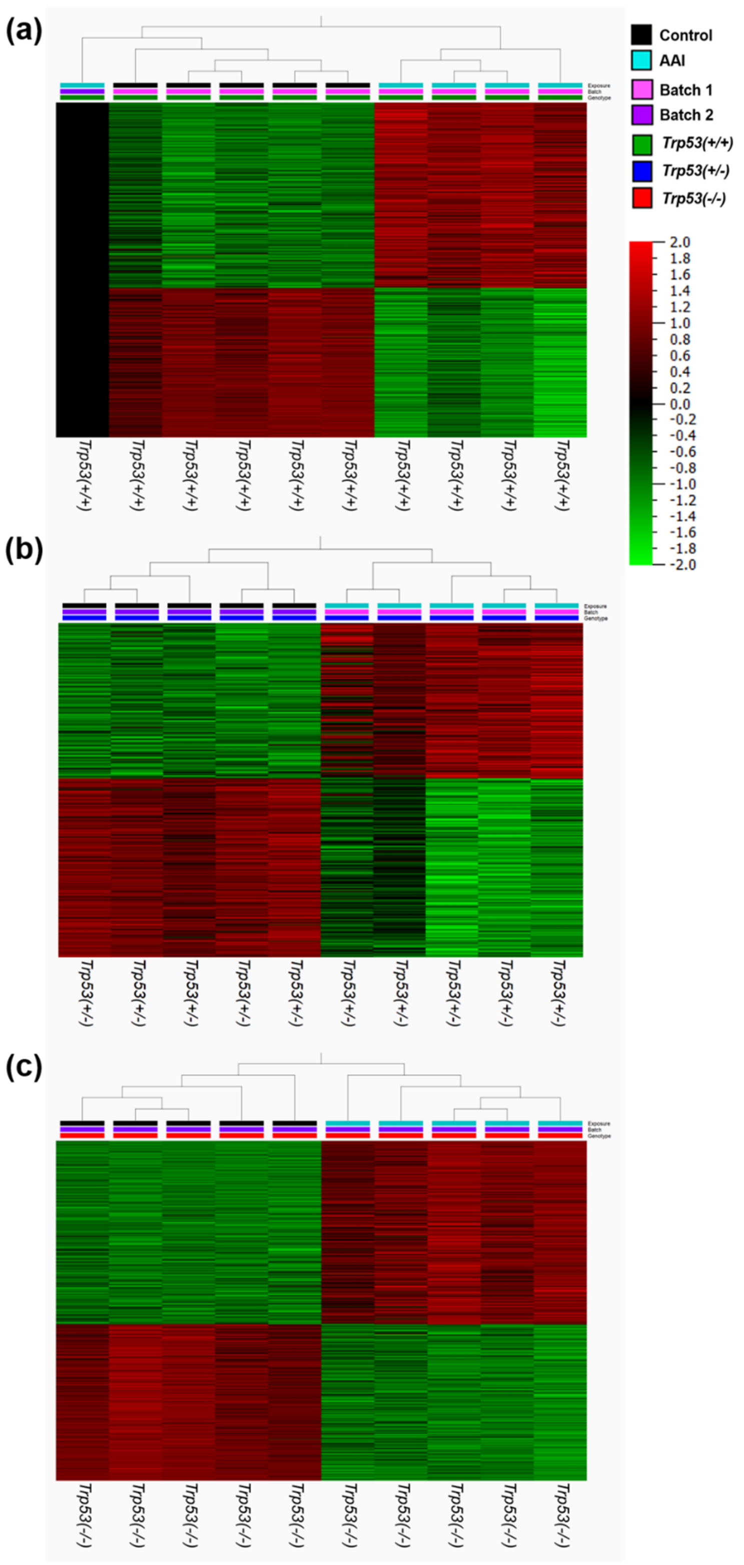

Figure. S1 The impact of *Trp53* genotype on AAI-induced gene expression *in vivo*. Hierarchical clustering and heat map of significantly altered (p<0.05; fold change ± 2) genes for AAI-exposed (**a**) *Trp53(+/+)*, (**b**) *Trp53(+/-)* and (**c**) *Trp53(-/-)* mouse kidneys. The heat map colours are based on gene expression (ordered in a decreasing manner for the AAI group), with red being up-regulated and green being down-regulated. Batches #1 and #2 indicate grouping of samples during cDNA synthesis.

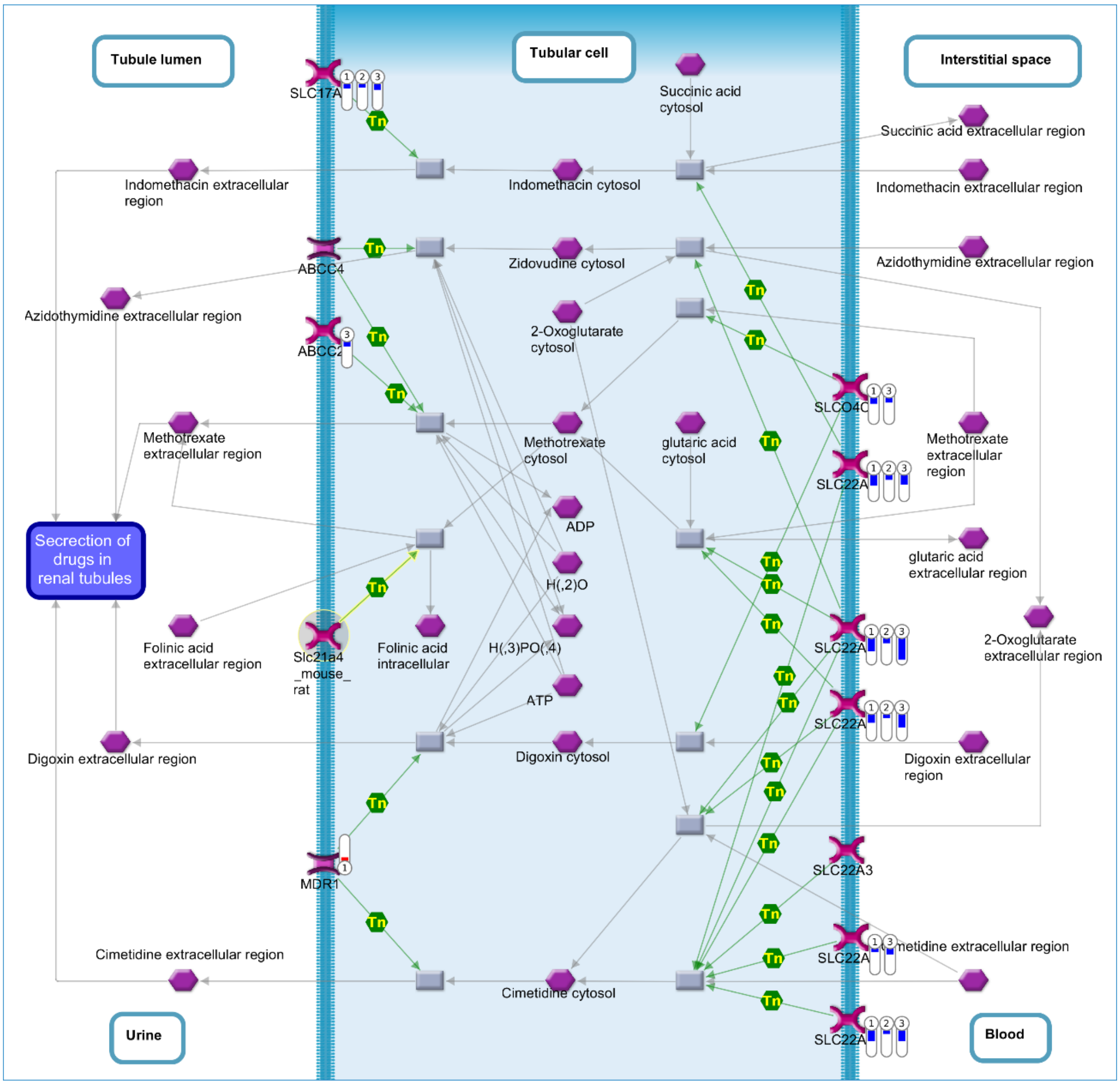

Figure. S2 Map of renal secretion of drugs in rodents. Significantly altered (p<0.05; fold change ± 2) genes for AAI-exposed *Trp53(+/+)*, *Trp53(+/-)* and *Trp53(-/-)* mouse kidneys were compared in MetaCore™. The enriched pathway (39th out of top 50; p<0.05) shows up-regulated (thermometer-like symbols in red) and down-regulated (thermometer-like symbols in blue) genes. Numbers indicate genotype: 1) *Trp53(+/+)*, 2) *Trp53(+/-)* and 3) *Trp53(-/-)*. For detailed legend see **Figure. S22**.

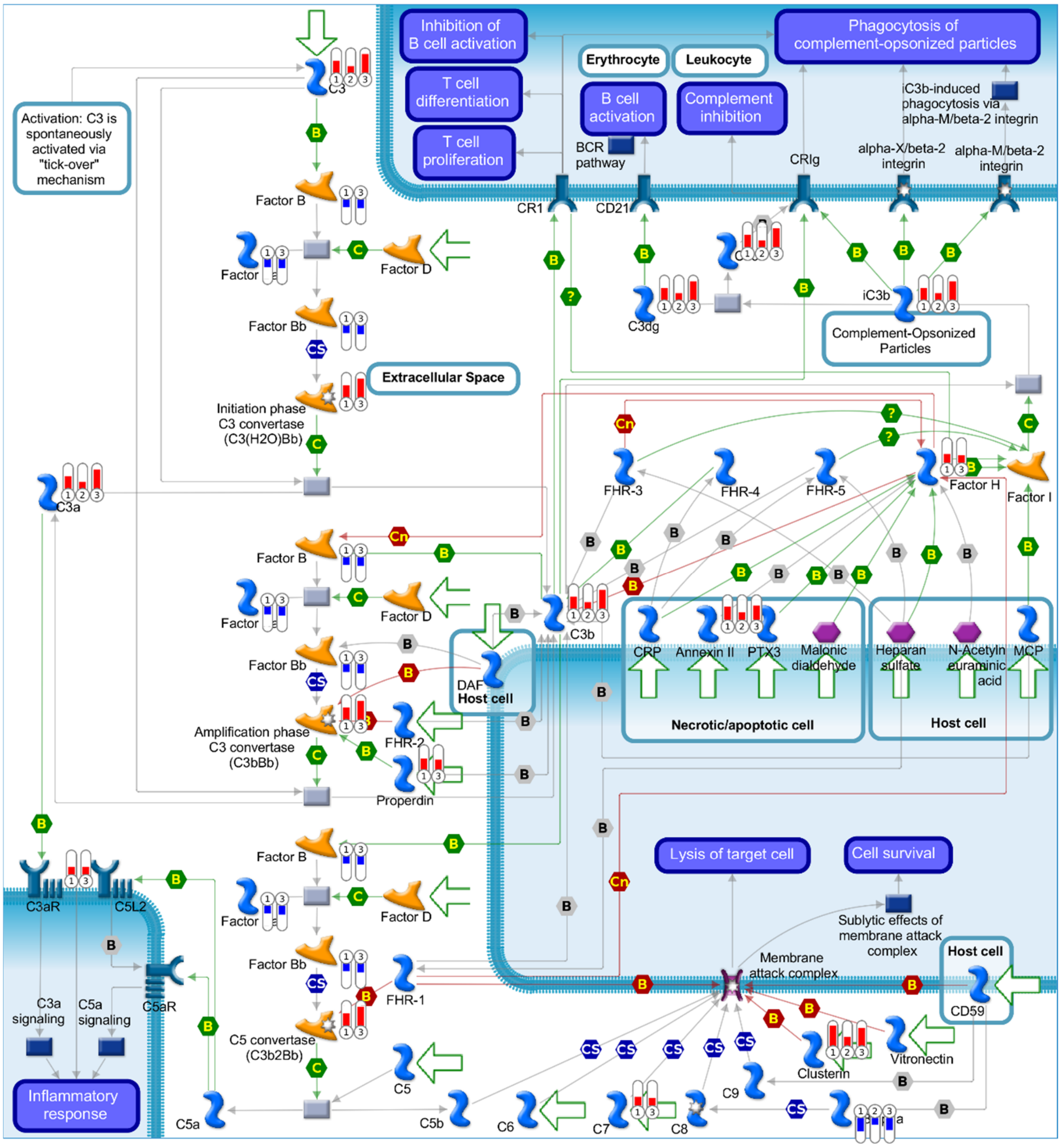

Figure. S3 Map of alternative complement pathway. Significantly altered (p<0.05; fold change ± 2) genes for AAI-exposed *Trp53(+/+)*, *Trp53(+/-)* and *Trp53(-/-)* mouse kidneys were compared in MetaCore™. The enriched pathway (3rd out of top 50; p<0.05) shows up-regulated (thermometer-like symbols in red) and down-regulated (thermometer-like symbols in blue) genes. Numbers indicate genotype: 1) *Trp53(+/+)*, 2) *Trp53(+/-)* and 3) *Trp53(-/-)*. For detailed legend see **Figure. S22**.

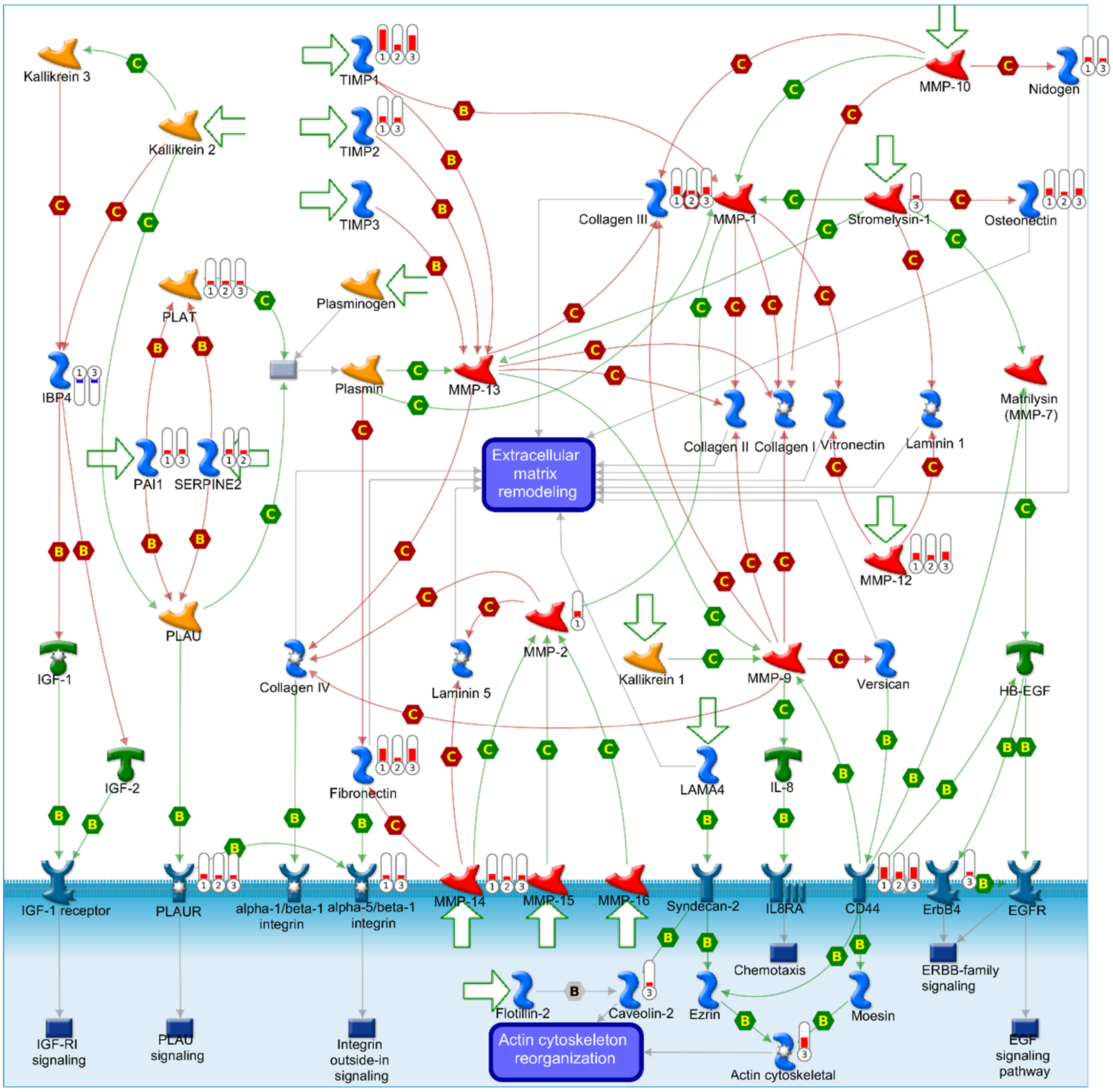

Figure. S4 Map of extracellular matrix remodelling. Significantly altered (p<0.05; fold change ± 2) genes for AAI-exposed *Trp53(+/+)*, *Trp53(+/-)* and *Trp53(-/-)* mouse kidneys were compared in MetaCore™. The enriched pathway (4th out of top 50; p<0.05) shows up-regulated (thermometer-like symbols in red) and down-regulated (thermometer-like symbols in blue) genes. Numbers indicate genotype: 1) *Trp53(+/+)*, 2) *Trp53(+/-)* and 3) *Trp53(-/-)*. For detailed legend see **Figure. S22**.

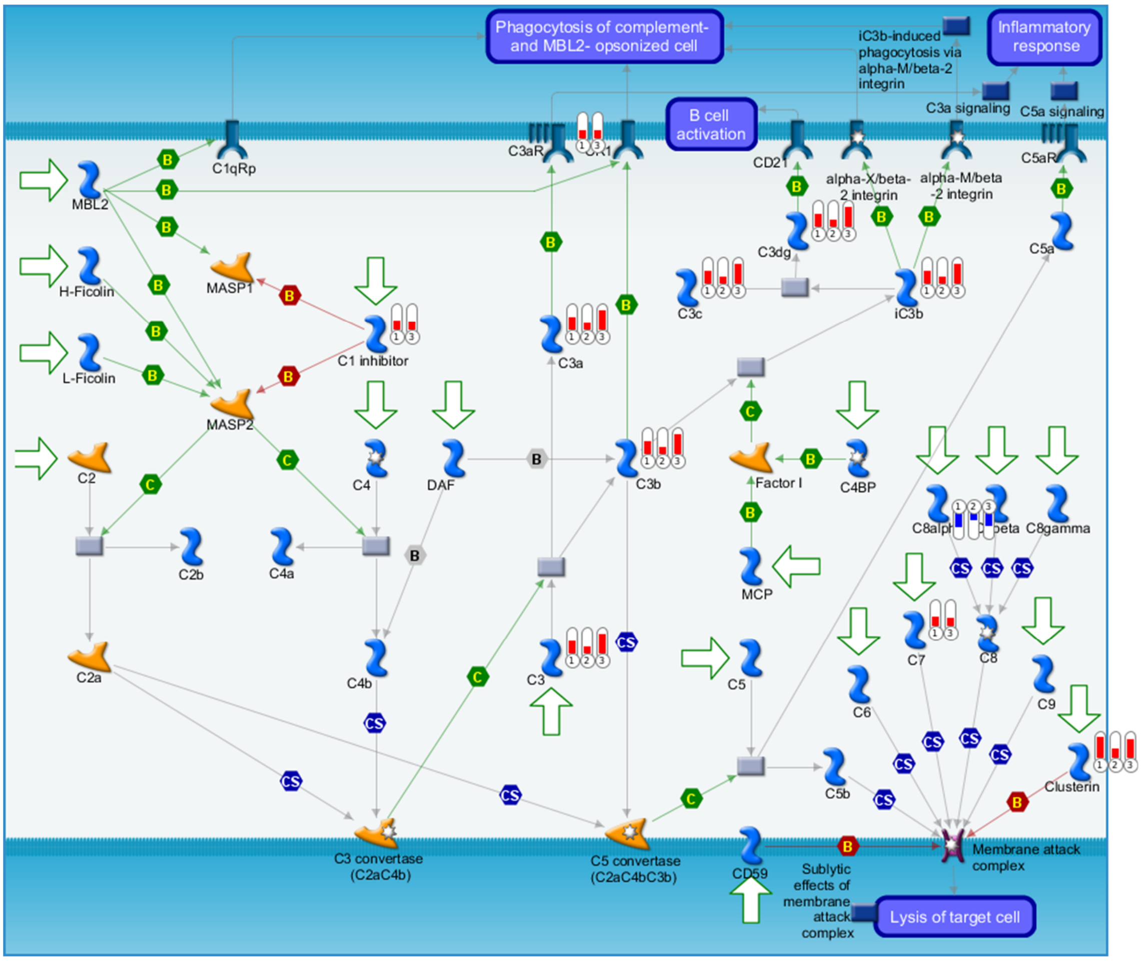

Figure. S5 Map of lectin-induced complement pathway. Significantly altered ($p<0.05$; fold change ± 2) genes for AAI-exposed *Trp53(+/+)*, *Trp53(+/-)* and *Trp53(-/-)* mouse kidneys were compared in MetaCore™. The enriched pathway (6th out of top 50; $p<0.05$) shows up-regulated (thermometer-like symbols in red) genes. Numbers indicate genotype: 1) *Trp53(+/+)*, 2) *Trp53(+/-)* and 3) *Trp53(-/-)*. For detailed legend see **Figure. S22**.

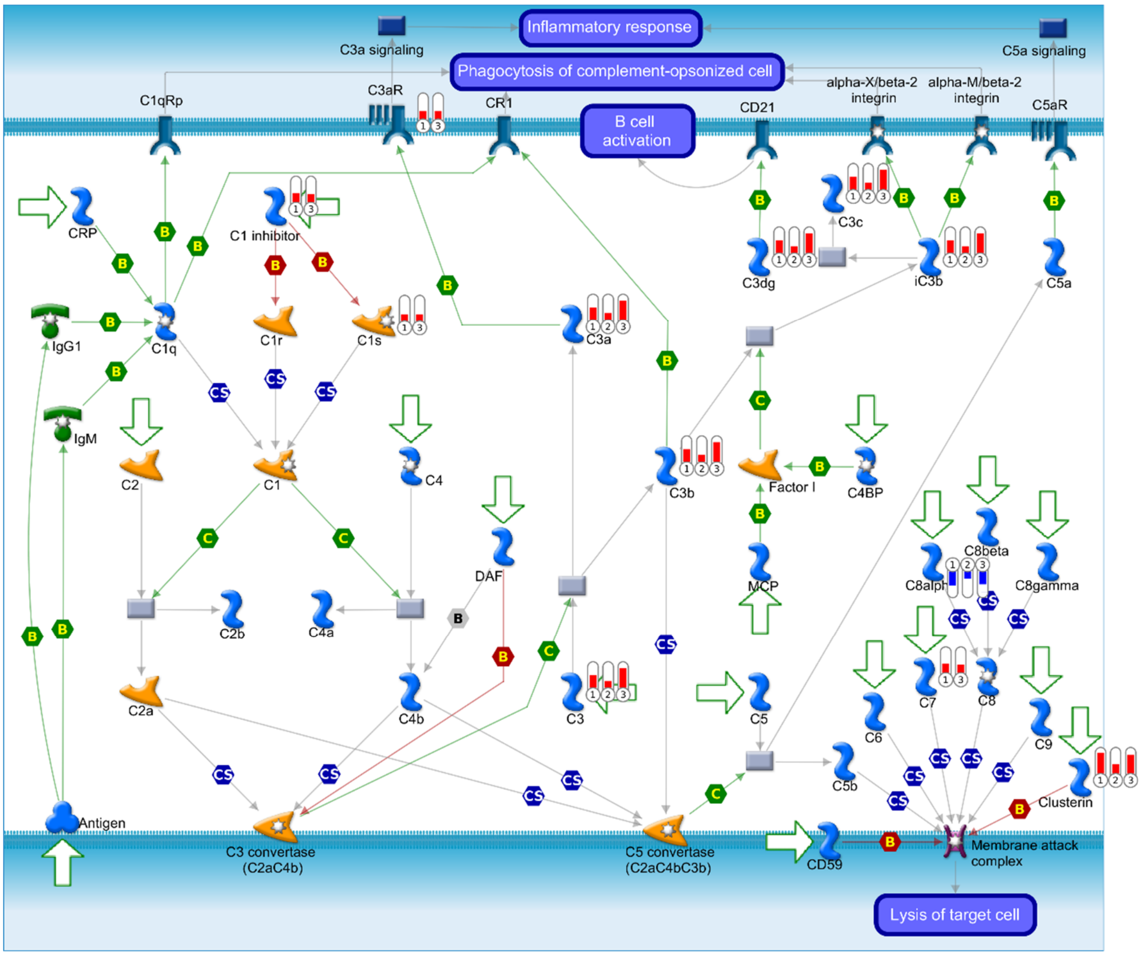

Figure. S6 Map of classical complement pathway. Significantly altered ($p<0.05$; fold change ± 2) genes for AAI-exposed *Trp53(+/+)*, *Trp53(+/-)* and *Trp53(-/-)* mouse kidneys were compared in MetaCore™. The enriched pathway (8th out of top 50; $p<0.05$) shows up-regulated (thermometer-like symbols in red) and down-regulated (thermometer-like symbols in blue) genes. Numbers indicate genotype: 1) *Trp53(+/+)*, 2) *Trp53(+/-)* and 3) *Trp53(-/-)*. For detailed legend see **Figure. S22**.

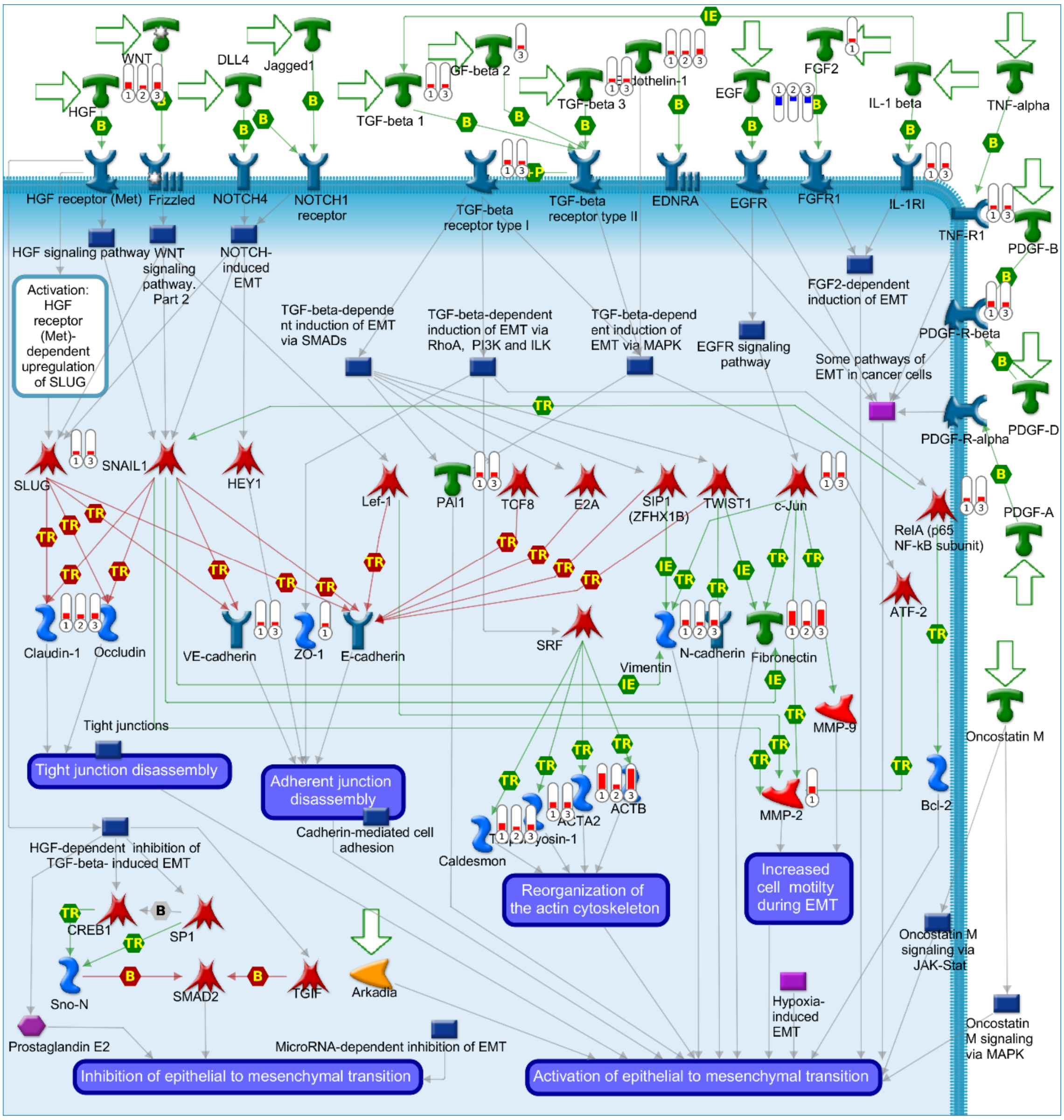

Figure. S7 Map of regulation of EMT. Significantly altered ($p<0.05$; fold change ± 2) genes for AAI-exposed *Trp53(+/+)*, *Trp53(+/-)* and *Trp53(-/-)* mouse kidneys were compared in MetaCore™. The enriched pathway (11th out of top 50; $p<0.05$) shows up-regulated (thermometer-like symbols in red) and down-regulated (thermometer-like symbols in blue) genes. Numbers indicate genotype: 1) *Trp53(+/+)*, 2) *Trp53(+/-)* and 3) *Trp53(-/-)*. For detailed legend see **Figure. S22**. Abbreviation: epithelial-to-mesenchymal transition (EMT).

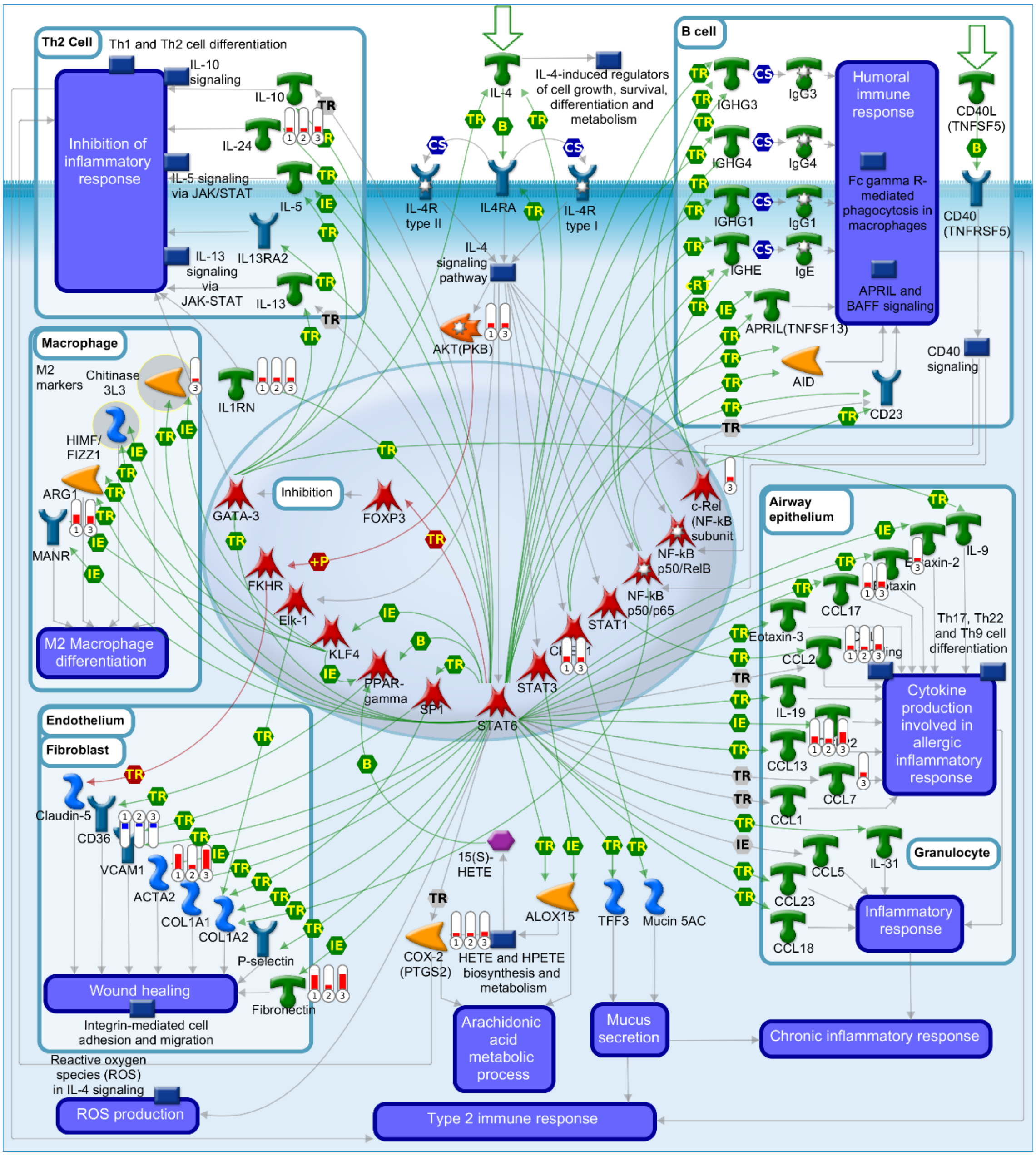

Figure. S8 Map of IL-4-responsive genes in type 2 immunity. Significantly altered (p<0.05; fold change ± 2) genes for AAI-exposed *Trp53(+/+)*, *Trp53(+/-)* and *Trp53(-/-)* mouse kidneys were compared in MetaCore™. The enriched pathway (13th out of top 50; p<0.05) shows up-regulated (thermometer-like symbols in red) and down-regulated (thermometer-like symbols in blue) genes. Numbers indicate genotype: 1) *Trp53(+/+)*, 2) *Trp53(+/-)* and 3) *Trp53(-/-)*. For detailed legend see **Figure. S22**. Abbreviation: interleukin-4 (IL-4).

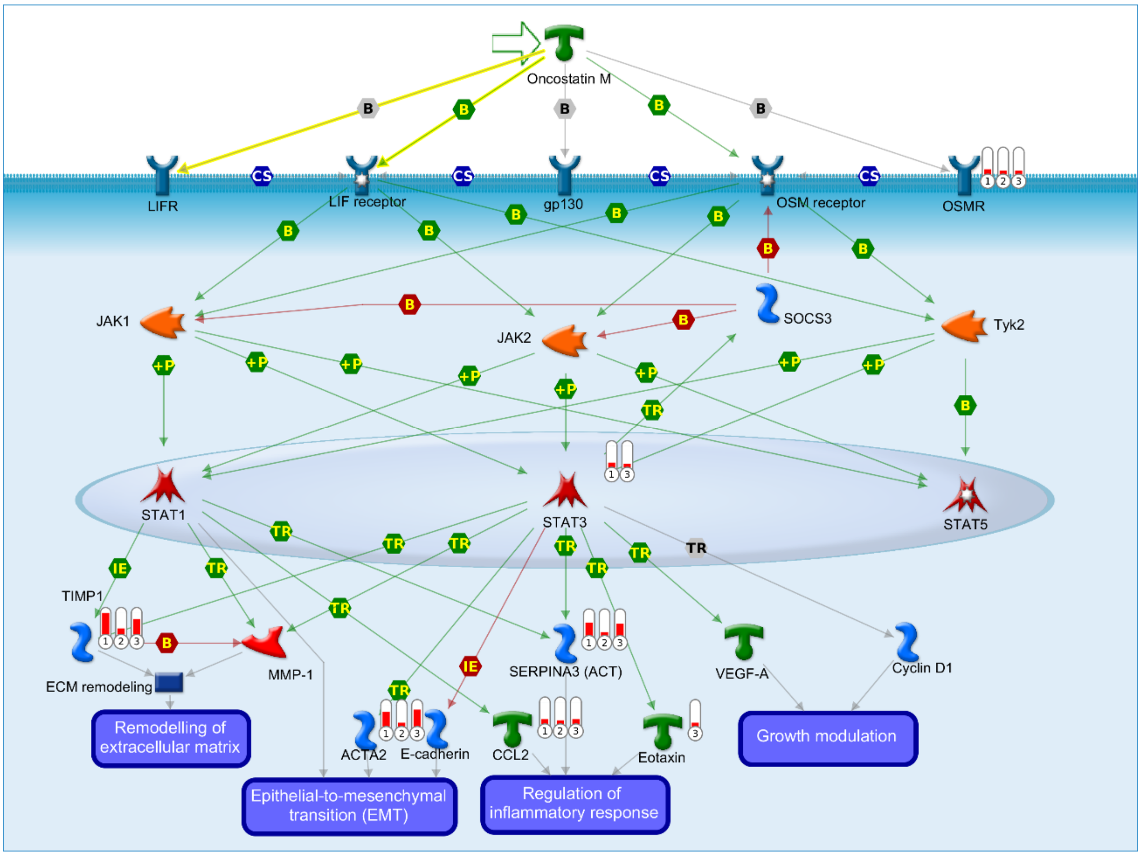

Figure. S9 Map of oncostatin M signalling via JAK-STAT. Significantly altered (p<0.05; fold change ± 2) genes for AAI-exposed *Trp53(+/+)*, *Trp53(+/-)* and *Trp53(-/-)* mouse kidneys were compared in MetaCore™. The enriched pathway (14th out of top 50; p<0.05) shows up-regulated (thermometer-like symbols in red) genes. Numbers indicate genotype: 1) *Trp53(+/+)*, 2) *Trp53(+/-)* and 3) *Trp53(-/-)*. For detailed legend see **Figure. S22**. Abbreviations: Janus kinase (JAK); signal transducer and activator of transcription (STAT).

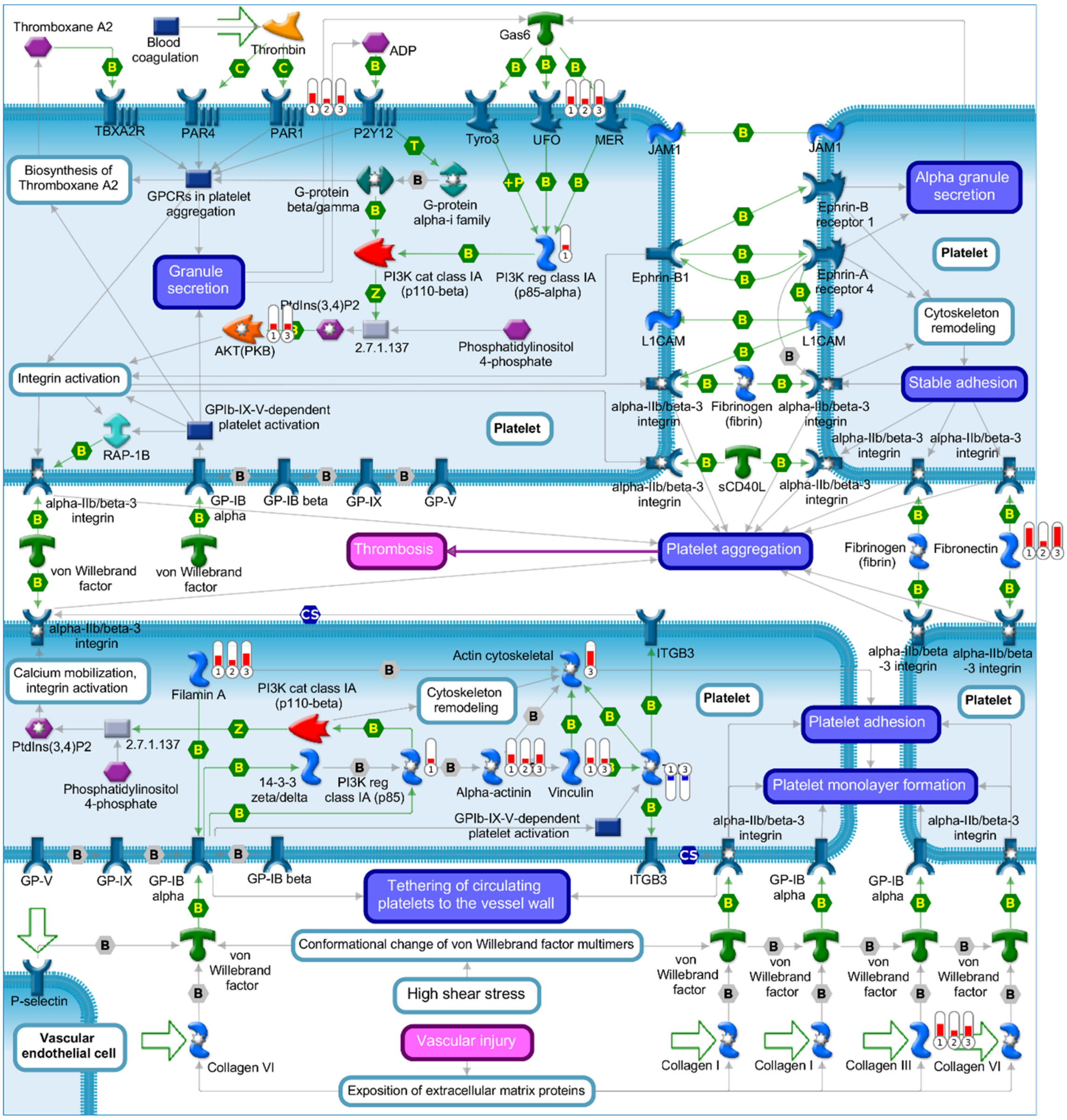

Figure. S10 Map of high shear stress-induced platelet activation. Significantly altered (p<0.05; fold change ± 2) genes for AAI-exposed *Trp53(+/+)*, *Trp53(+/-)* and *Trp53(-/-)* mouse kidneys were compared in MetaCore™. The enriched pathway (18th out of top 50; p<0.05) shows up-regulated (thermometer-like symbols in red) and down-regulated (thermometer-like symbols in blue) genes. Numbers indicate genotype: 1) *Trp53(+/+)*, 2) *Trp53(+/-)* and 3) *Trp53(-/-)*. For detailed legend see **Figure. S22**.

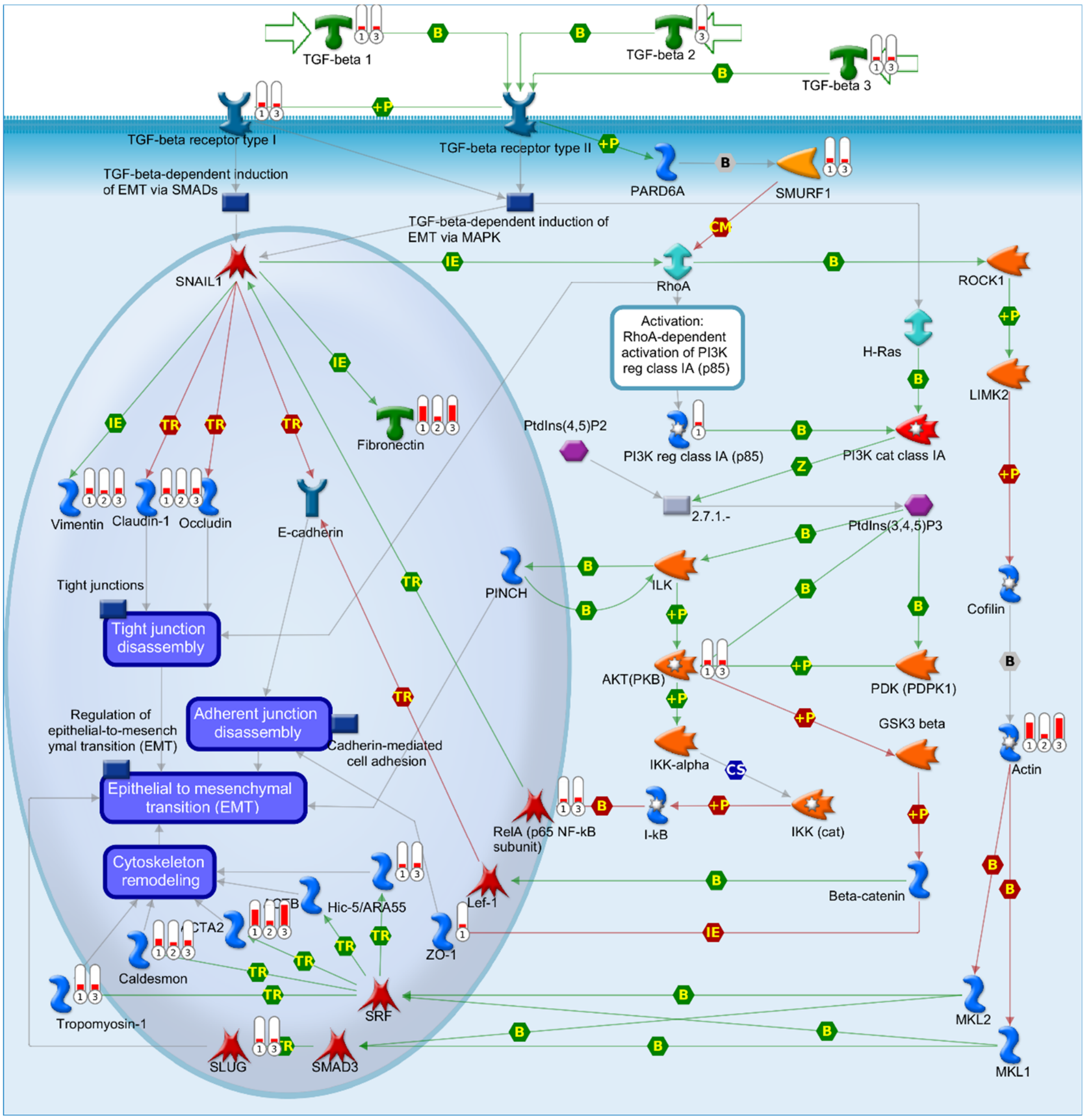

Figure. S11 Map of TGF-β-dependent induction of EMT via RhoA, PI3K and ILK. Significantly altered (*p*<0.05; fold change ± 2) genes for AAI-exposed *Trp53(+/+)*, *Trp53(+/-)* and *Trp53(-/-)* mouse kidneys were compared in MetaCore™. The enriched pathway (19th out of top 50; *p*<0.05) shows up-regulated (thermometer-like symbols in red) genes. Numbers indicate genotype: 1) *Trp53(+/+)*, 2) *Trp53(+/-)* and 3) *Trp53(-/-)*. For detailed legend see **Figure. S22**. Abbreviations: epithelial-to-mesenchymal transition (EMT); integrin-linked protein kinase (ILK); phosphoinositide 3-kinase (PI3K); *RAS* homolog gene family, member A (RhoA); transforming growth factor β (TGF-β).

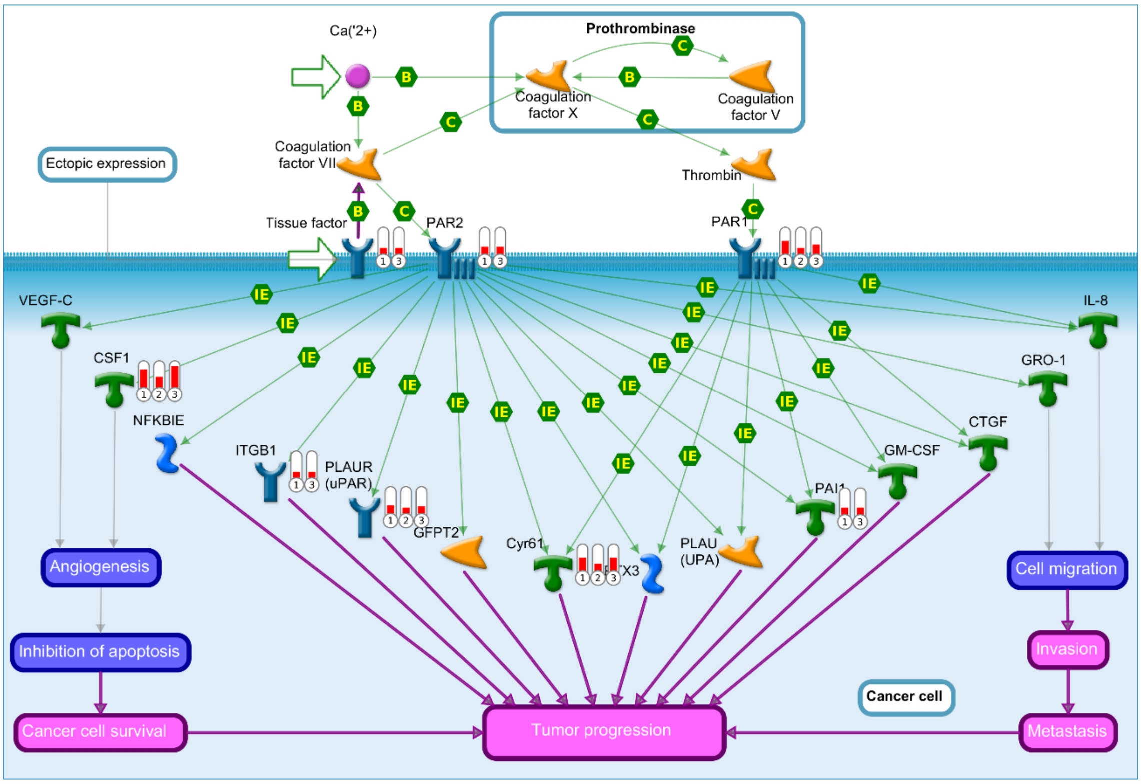

Figure. S12 Map of tissue factor signalling in cancer. Significantly altered (p<0.05; fold change ± 2) genes for AAI-exposed *Trp53(+/+)*, *Trp53(+/-)* and *Trp53(-/-)* mouse kidneys were compared in MetaCore™. The enriched pathway (26th out of top 50; p<0.05) shows up-regulated (thermometer-like symbols in red) genes. Numbers indicate genotype: 1) *Trp53(+/+)*, 2) *Trp53(+/-)* and 3) *Trp53(-/-)*. For detailed legend see **Figure. S22**.

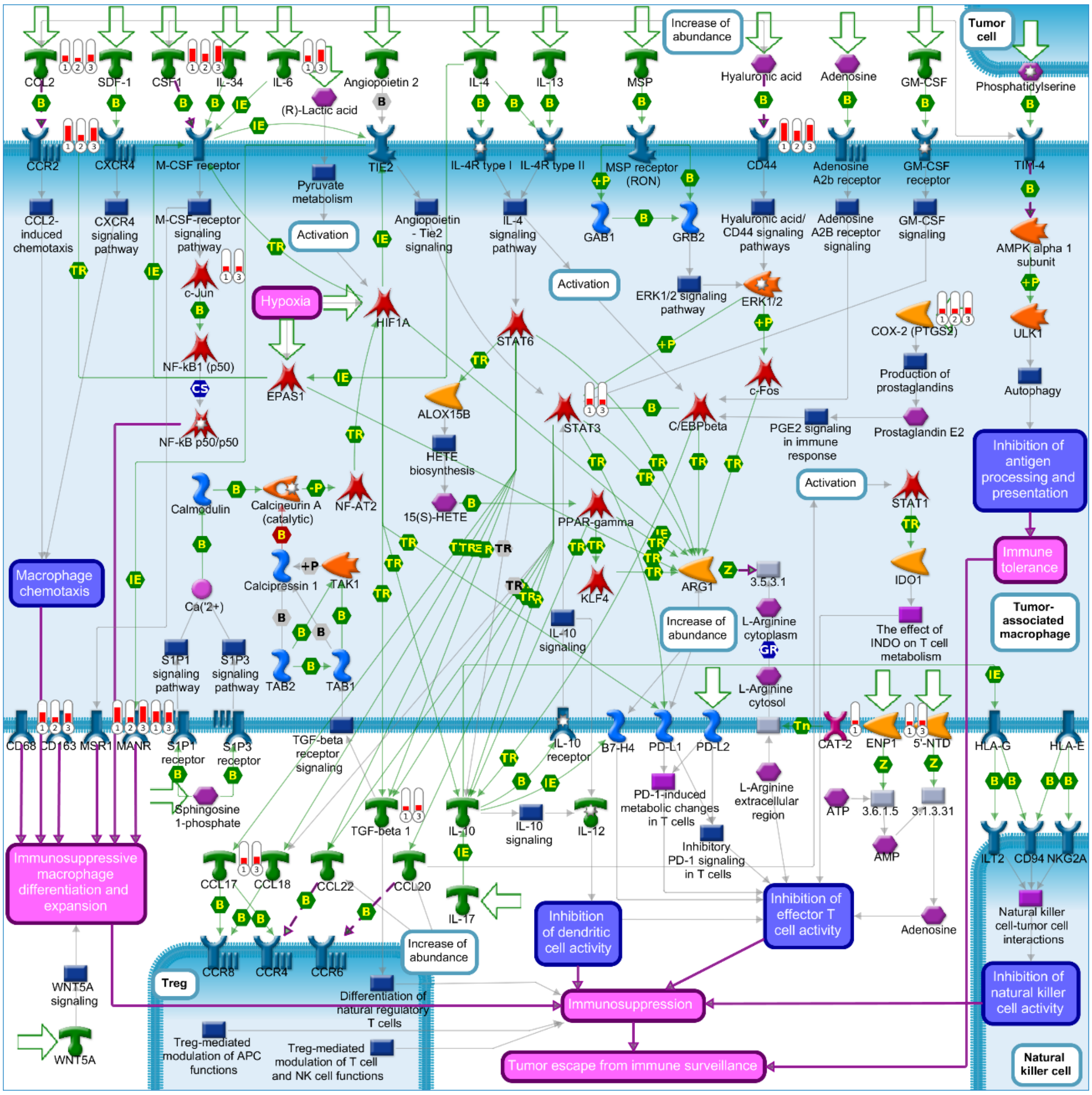

Figure. S13 Map of macrophage-induced immunosuppression in the tumour microenvironment. Significantly altered (p<0.05; fold change ± 2) genes for AAI-exposed *Trp53(+/+)*, *Trp53(+/-)* and *Trp53(-/-)* mouse kidneys were compared in MetaCore™. The enriched pathway (36th out of top 50; p<0.05) shows up-regulated (thermometer-like symbols in red) genes. Numbers indicate genotype: 1) *Trp53(+/+)*, 2) *Trp53(+/-)* and 3) *Trp53(-/-)*. For detailed legend see **Figure. S22**.

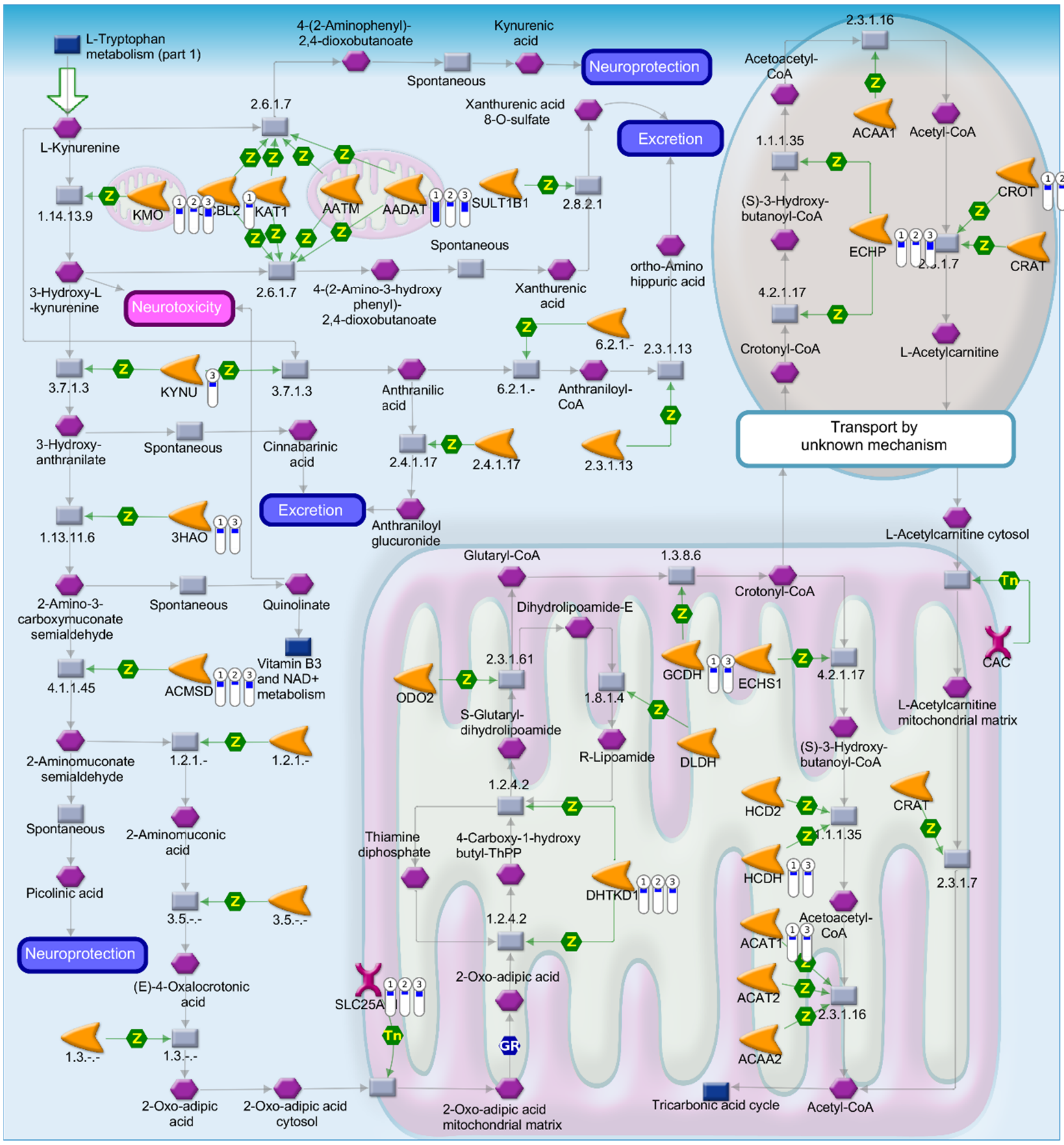

Figure. S14 Map of L-Tryptophan metabolism. Significantly altered (p<0.05; fold change ± 2) genes for AAI-exposed *Trp53(+/+)*, *Trp53(+/-)* and *Trp53(-/-)* mouse kidneys were compared in MetaCore™. The enriched pathway (37th out of top 50; p<0.05) shows down-regulated (thermometer-like symbols in blue) genes. Numbers indicate genotype: 1) *Trp53(+/+)*, 2) *Trp53(+/-)* and 3) *Trp53(-/-)*. For detailed legend see **Figure. S22**.

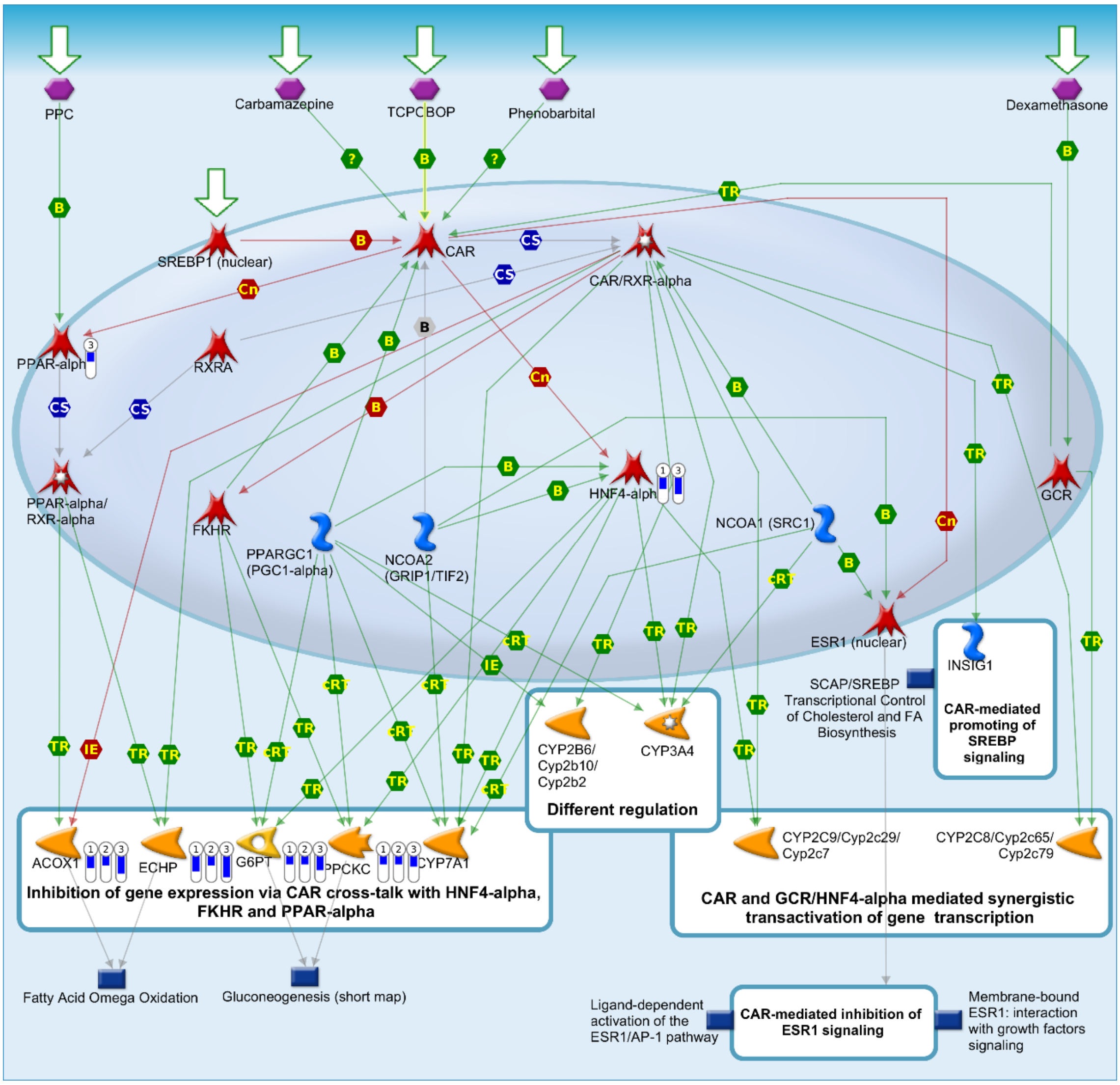

Figure. S15 Map of CAR signalling via cross-talk in rodents. Significantly altered (p<0.05; fold change ± 2) genes for AAI-exposed *Trp53(+/+)*, *Trp53(+/-)* and *Trp53(-/-)* mouse kidneys were compared in MetaCore™. The enriched pathway (38th out of top 50; p<0.05) shows down-regulated (thermometer-like symbols in blue) genes. Numbers indicate genotype: 1) *Trp53(+/+)*, 2) *Trp53(+/-)* and 3) *Trp53(-/-)*. For detailed legend see **Figure. S22**. Abbreviation: constitutive androstane receptor (CAR).

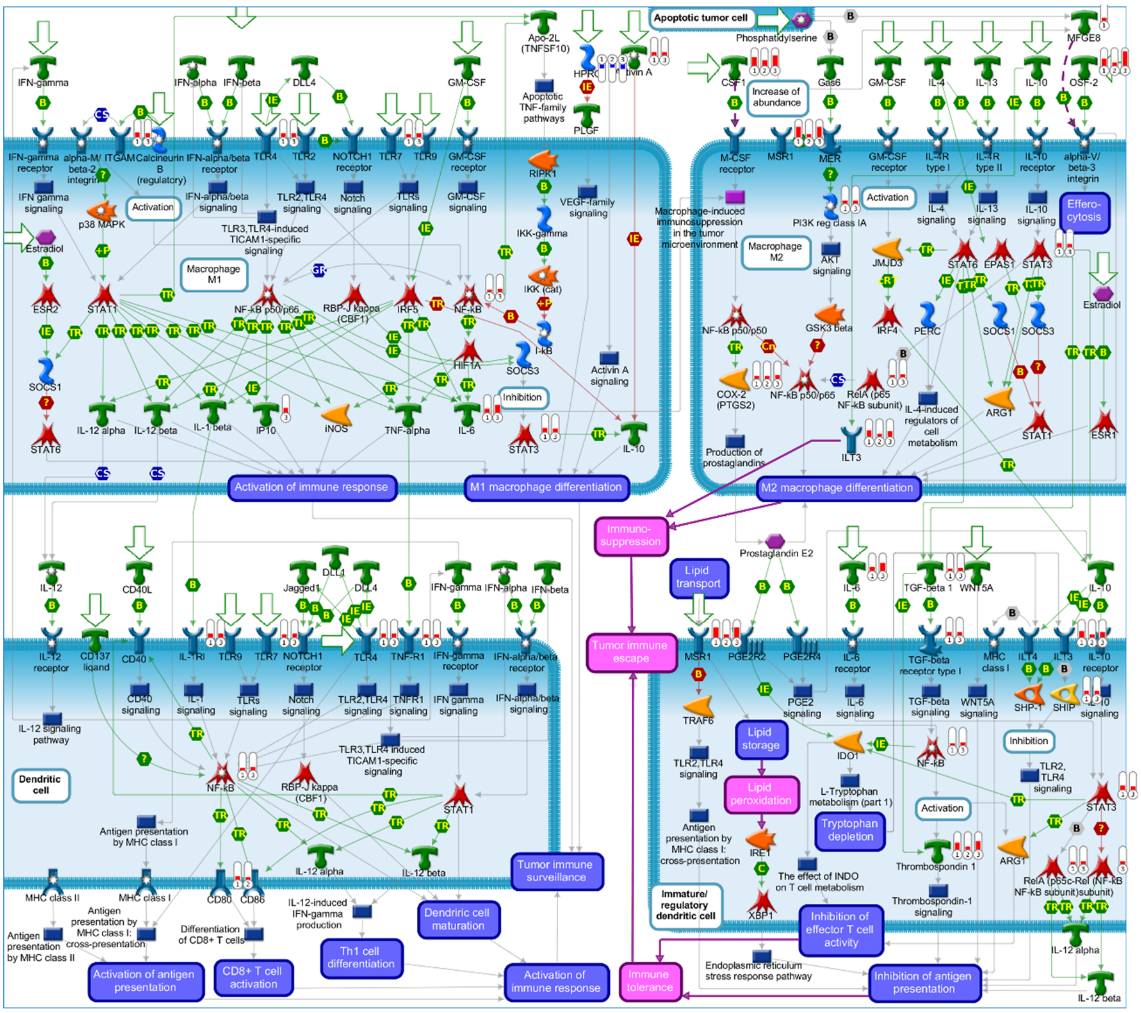

Figure. S16 Map of macrophage and dendritic cell phenotype shift in cancer. Significantly altered (p<0.05; fold change ± 2) genes for AAI-exposed *Trp53(+/+)*, *Trp53(+/-)* and *Trp53(-/-)* mouse kidneys were compared in MetaCore™. The enriched pathway (41st out of top 50; p<0.05) shows up-regulated (thermometer-like symbols in red) and down-regulated (thermometer-like symbols in blue) genes. Numbers indicate genotype: 1) *Trp53(+/+)*, 2) *Trp53(+/-)* and 3) *Trp53(-/-)*. For detailed legend see **Figure. S22**.

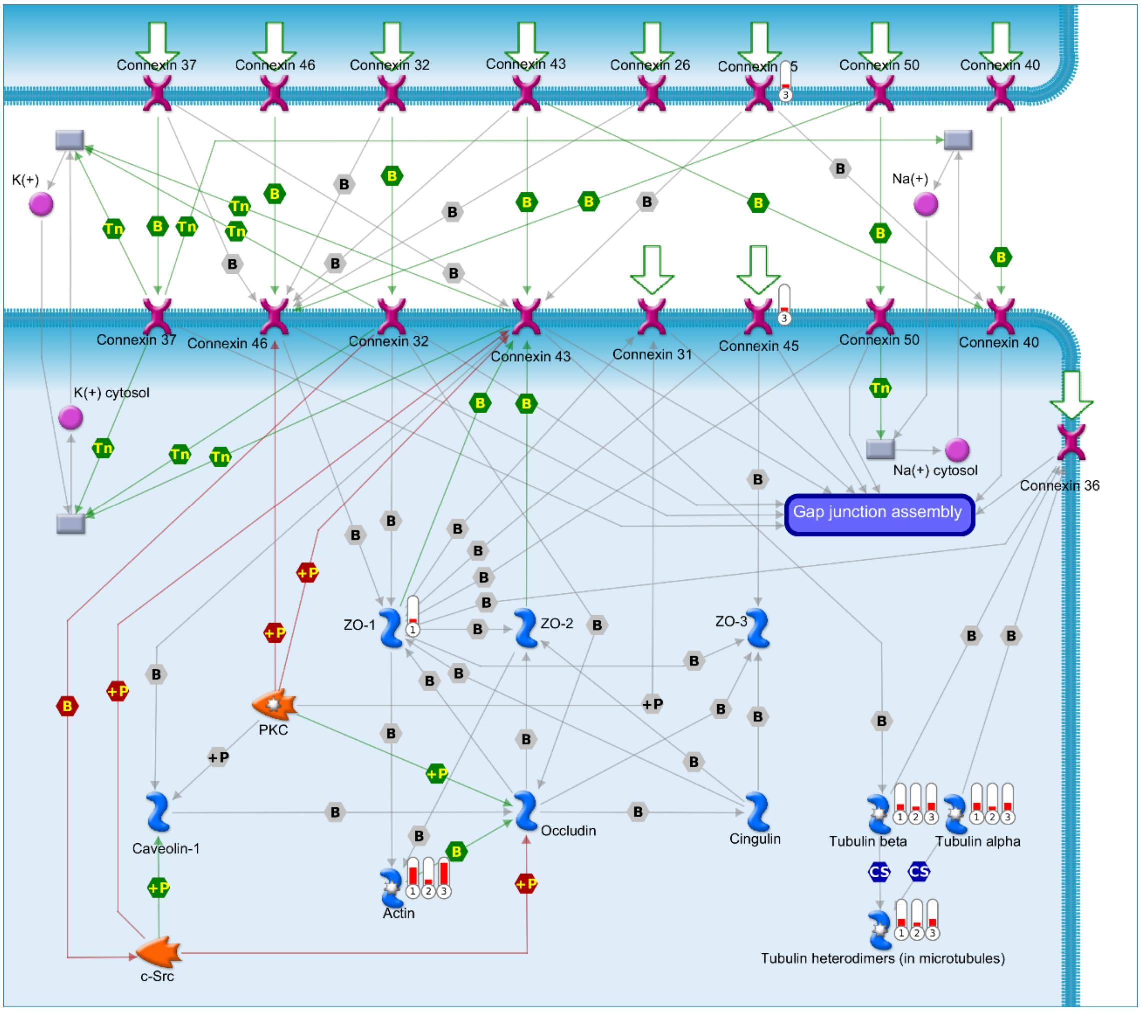

Figure. S17 Map of gap junctions. Significantly altered (*p*<0.05; fold change ± 2) genes for AAI-exposed *Trp53(+/+)*, *Trp53(+/-)* and *Trp53(-/-)* mouse kidneys were compared in MetaCore™. The enriched pathway (44th out of top 50; *p*<0.05) shows up-regulated (thermometer-like symbols in red) genes. Numbers indicate genotype: 1) *Trp53(+/+)*, 2) *Trp53(+/-)* and 3) *Trp53(-/-)*. For detailed legend see **Figure. S22**.

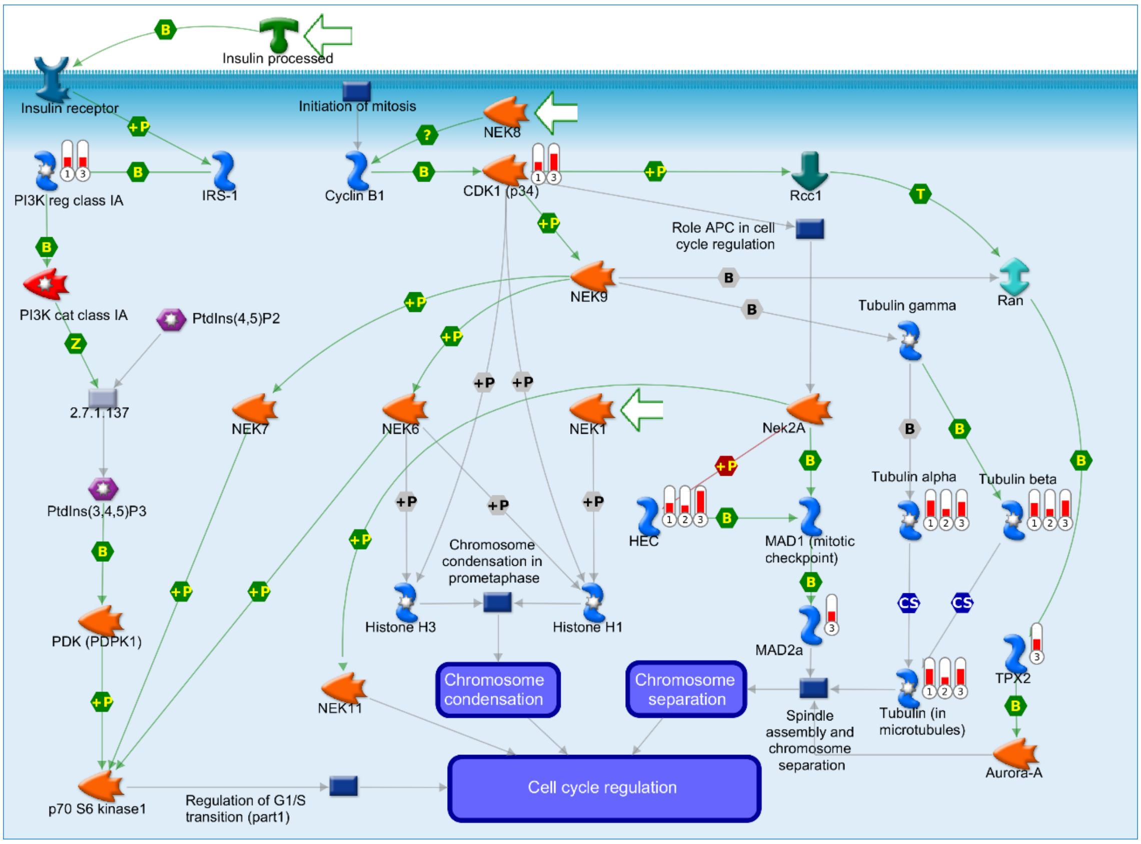

Figure. S18 Map of role of NEK in cell cycle regulation. Significantly altered (p<0.05; fold change ± 2) genes for AAI-exposed *Trp53(+/+)*, *Trp53(+/-)* and *Trp53(-/-)* mouse kidneys were compared in MetaCore™. The enriched pathway (47th out of top 50; p<0.05) shows up-regulated (thermometer-like symbols in red) genes. Numbers indicate genotype: 1) *Trp53(+/+)*, 2) *Trp53(+/-)* and 3) *Trp53(-/-)*. For detailed legend see **Figure. S22**. Abbreviation: never in mitosis, gene A-related kinase (NEK).

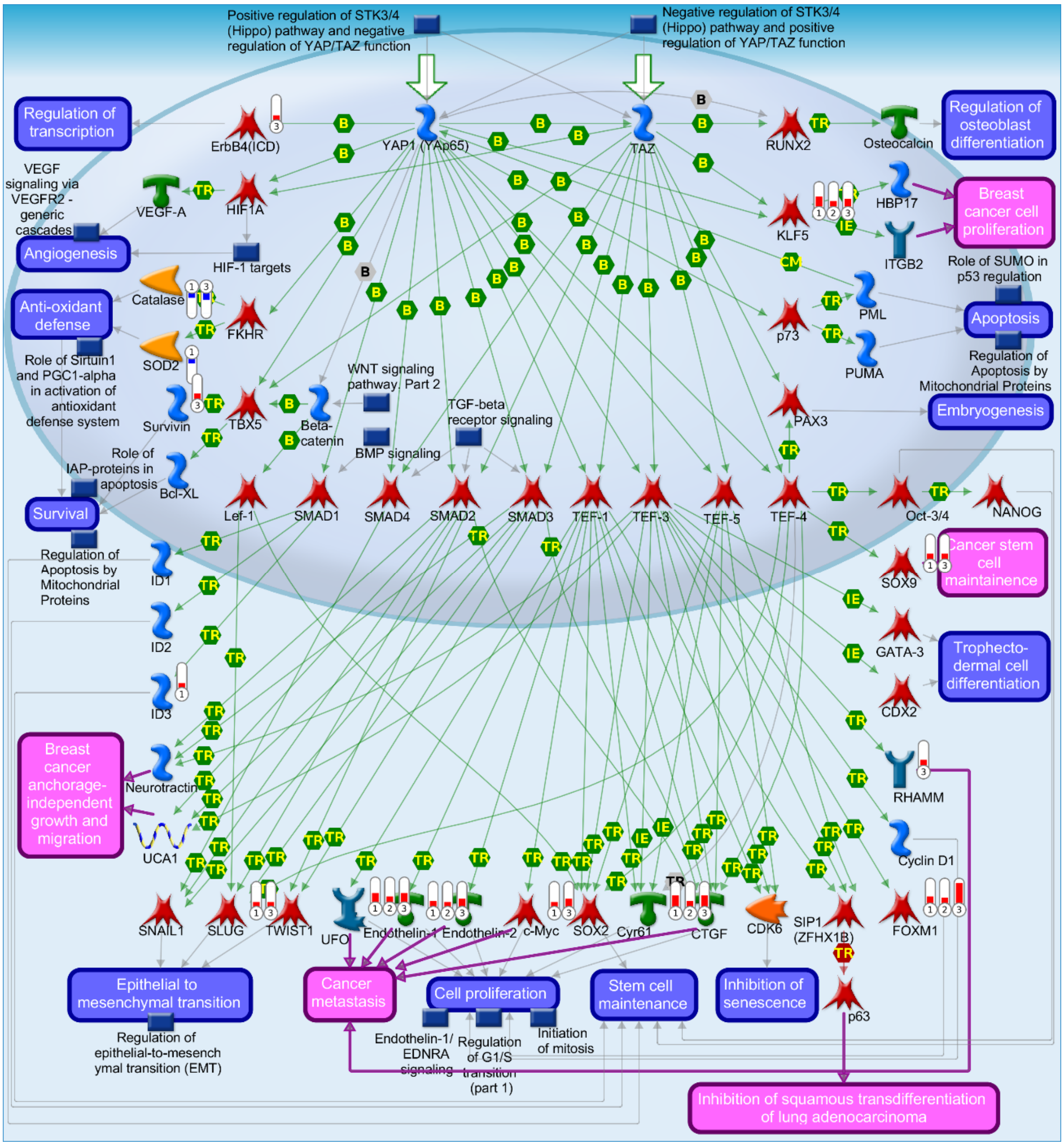

Figure. S19 Map of YAP/TAZ-mediated co-regulation of transcription. Significantly altered (p<0.05; fold change ± 2) genes for AAI-exposed *Trp53(+/+)*, *Trp53(+/-)* and *Trp53(-/-)* mouse kidneys were compared in MetaCore™. The enriched pathway (49th out of top 50; p<0.05) shows up-regulated (thermometer-like symbols in red) and down-regulated (thermometer-like symbols in blue) genes. Numbers indicate genotype: 1) *Trp53(+/+)*, 2) *Trp53(+/-)* and 3) *Trp53(-/-)*. For detailed legend see **Figure. S22**. Abbreviations: Tafazzin (TAZ); yes-associated protein (YAP).

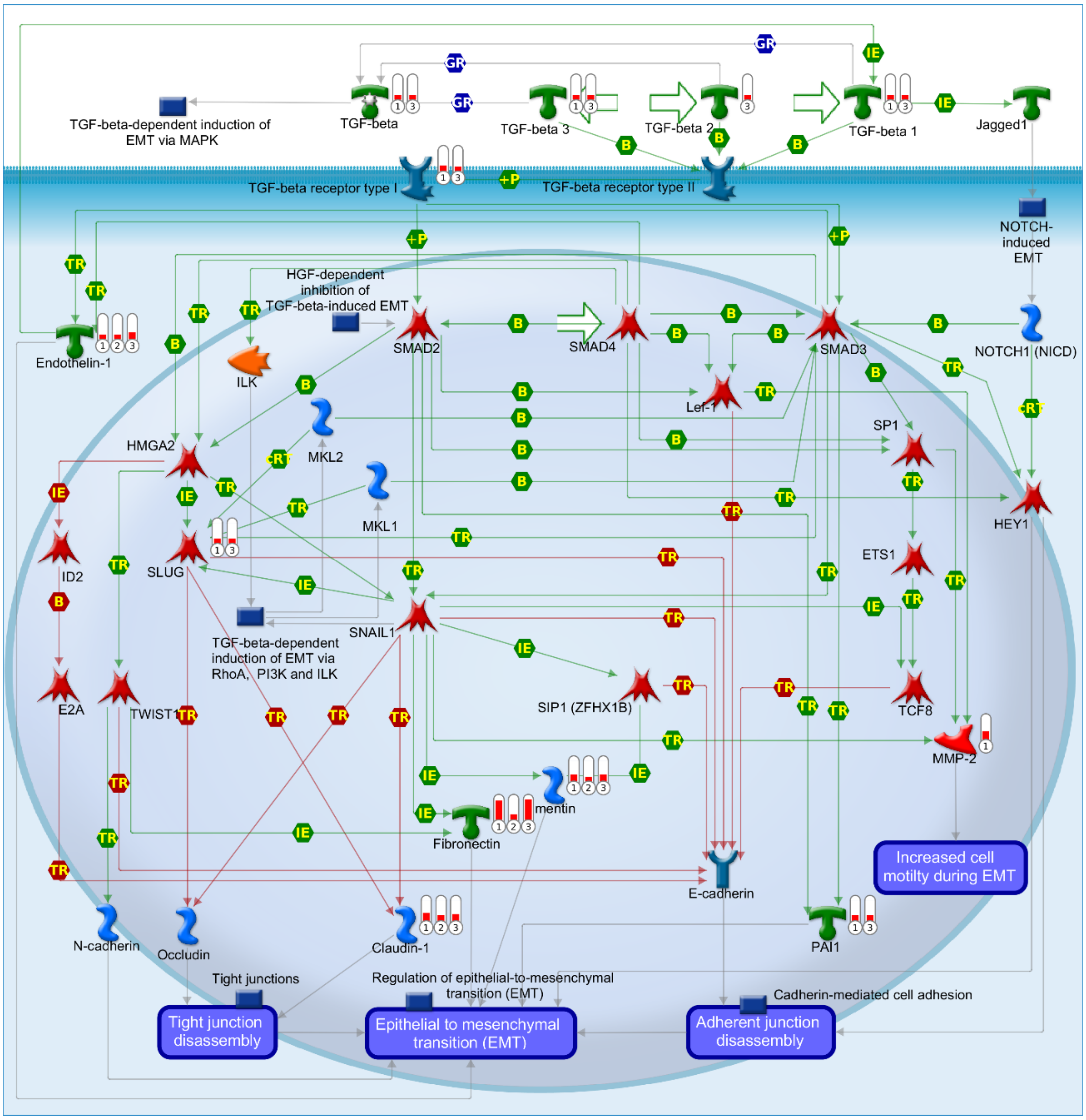

Figure. S20 Map of TGF-β-dependent induction of EMT via SMADs. Significantly altered (*p*<0.05; fold change ± 2) genes for AAI-exposed *Trp53(+/+)*, *Trp53(+/-)* and *Trp53(-/-)* mouse kidneys were compared in MetaCore™. The enriched pathway (50th out of top 50; *p*<0.05) shows up-regulated (thermometer-like symbols in red) genes. Numbers indicate genotype: 1) *Trp53(+/+)*, 2) *Trp53(+/-)* and 3) *Trp53(-/-)*. For detailed legend see **Figure. S22**. Abbreviations: epithelial-to-mesenchymal transition (EMT); *Sma* mothers against DPP homology (SMAD); transforming growth factor β (TGF-β).

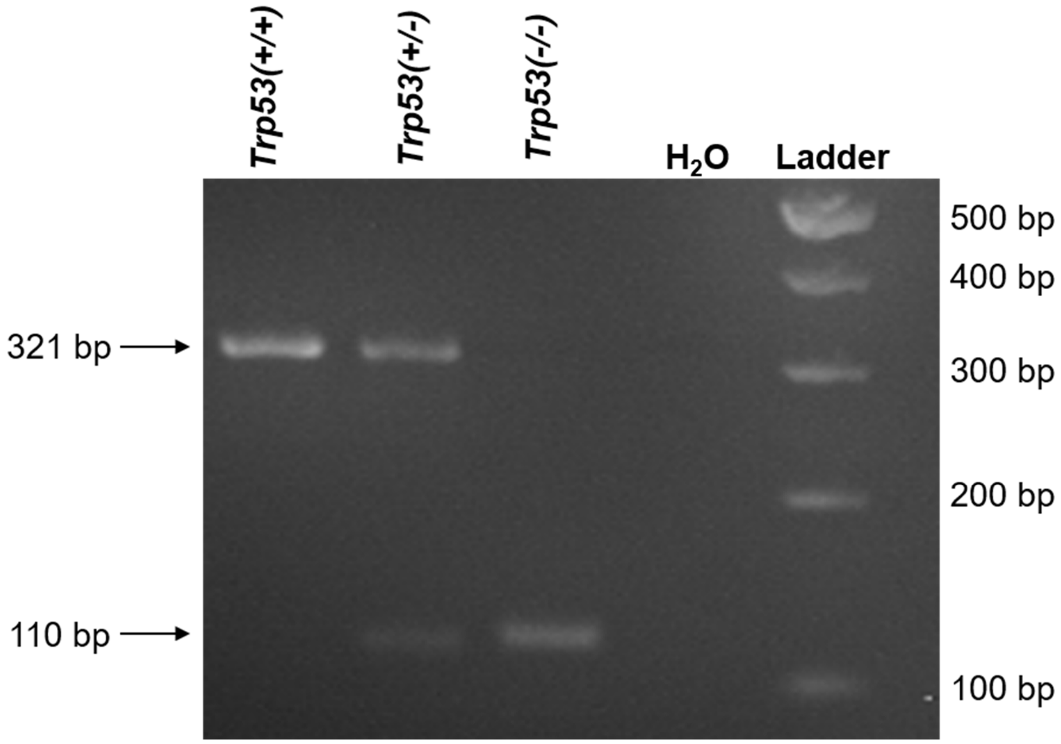

Figure. S21 Example of a *Trp53(+/+)*, *Trp53(+/-)* and *Trp53(-/-)* mouse genotyping PCR. H_2O indicates the negative control. bp, base pairs.

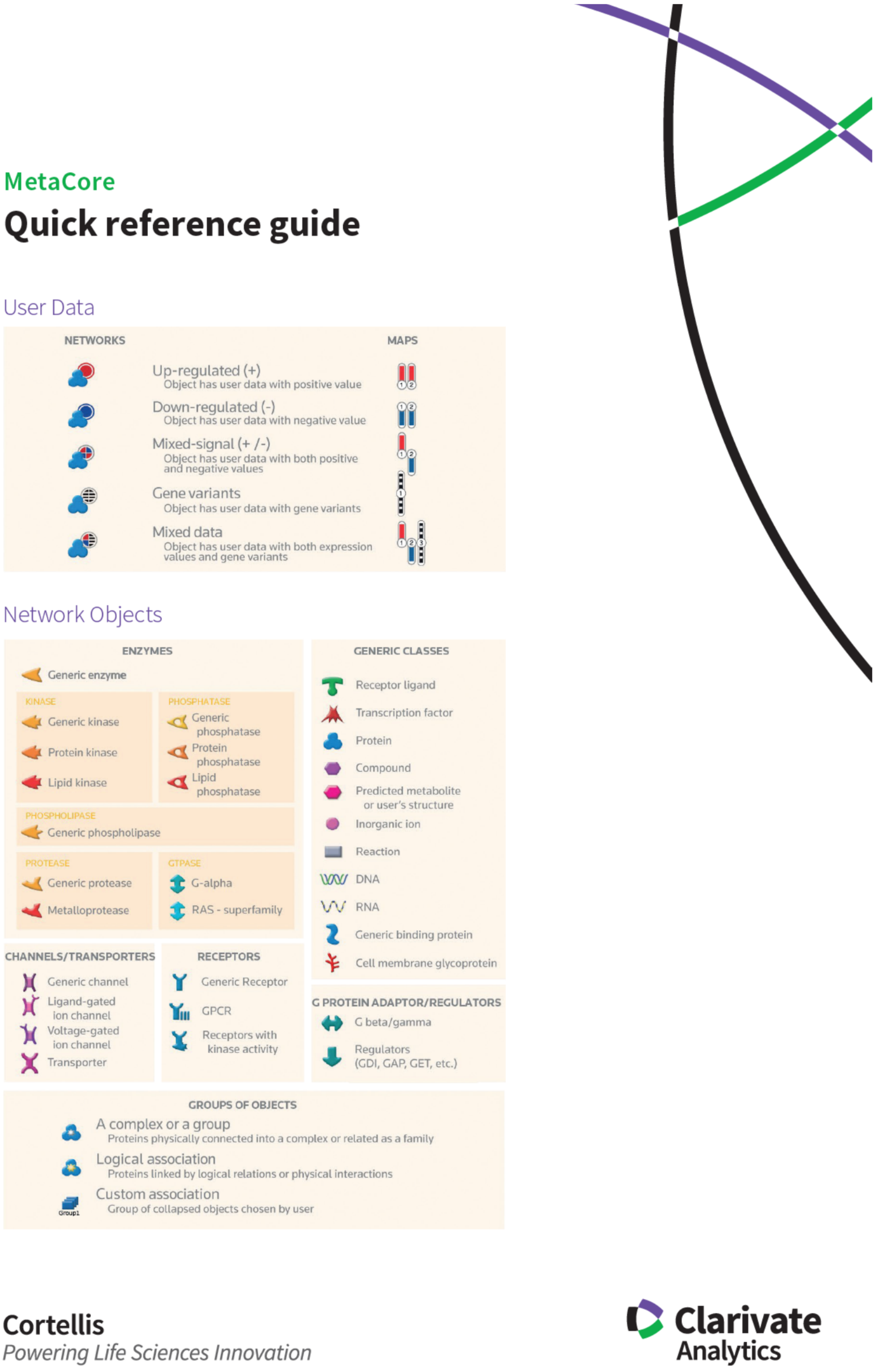

Figure. S22 MetaCore™ "Quick reference guide" (Part 1).

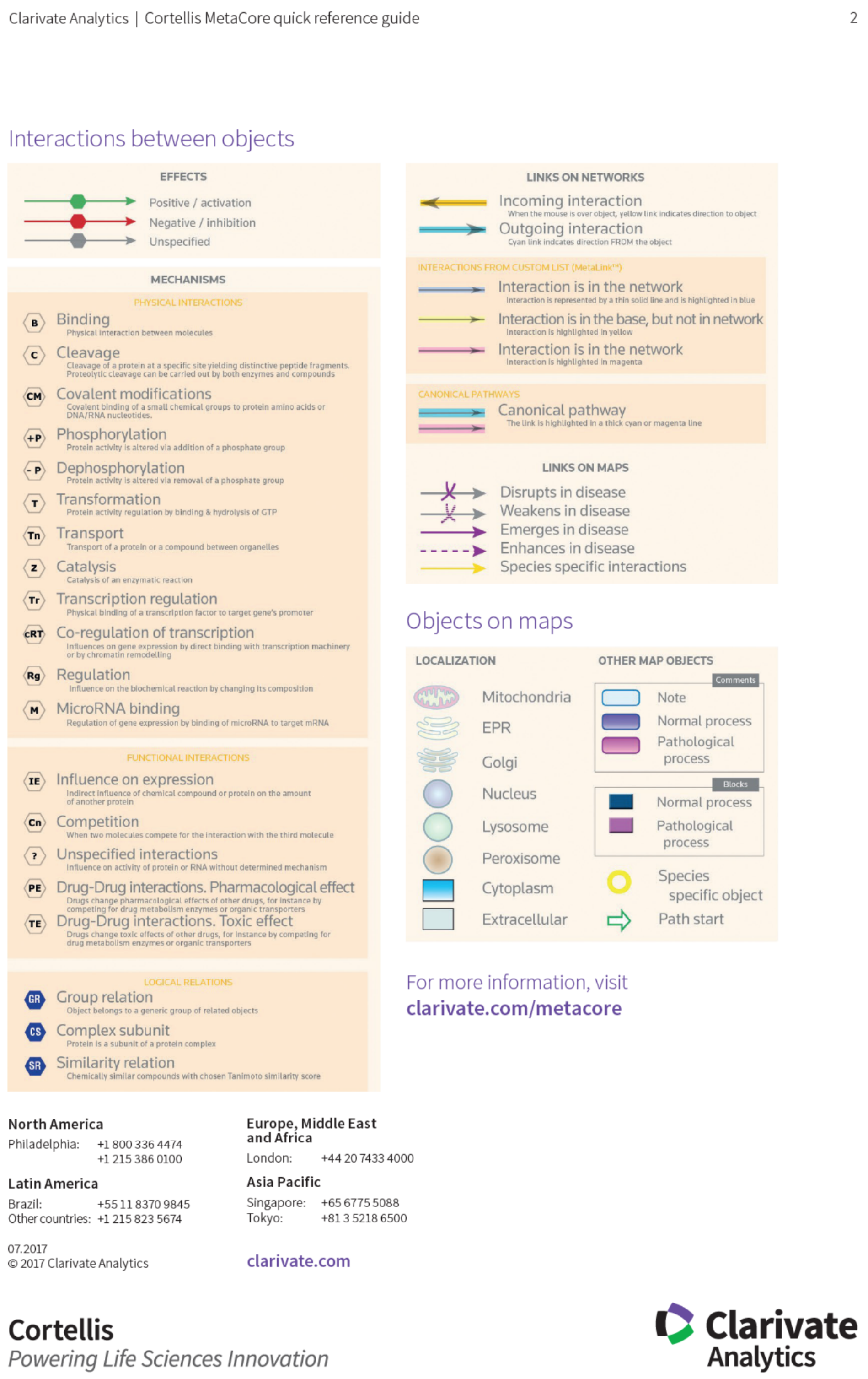

Figure. S22 MetaCore™ "Quick reference guide" (Part 2).
